# *Candida* spp. suppress neutrophil reactive nitrogen species to evade killing

**DOI:** 10.1128/mbio.00202-26

**Published:** 2026-07-06

**Authors:** Thomas B. Burgess, Ffion R. Hammond, Piotr T. Szkuta, Amy Lewis, Stella Christou, Keiran A. Bowden, Tihana Bicanic, Lynne R. Prince, Kathryn R. Ayscough, David G. Partridge, Simon A. Johnston, Alison M. Condliffe, Philip M. Elks

**Affiliations:** 1 Bateson Centre for Disease Mechanisms and Florey Institute of Infection, School of Medicine and Population Health, The University of Sheffield 12210, Sheffield, United Kingdom; 2 The School of Biosciences, The University of Sheffield 152813, Sheffield, England, United Kingdom; 3 Institute for Infection and Immunity, City St George's University of London 244882, London, United Kingdom; 4 Department of Microbiology, Laboratory Medicine, Northern General Hospital, Sheffield Teaching Hospital NHS Foundation Trust 575008, Sheffield, United Kingdom; Texas Christian University, Fort Worth, Texas, USA

**Keywords:** *Candida albicans*, *Candida*, neutrophils, innate immunity, zebrafish

## Abstract

*Candida albicans* is a human commensal that can cause life-threatening invasive infection in immunocompromised individuals. Human immunity to *C. albicans* infection is thought to be largely dependent on neutrophil reactive oxygen and nitrogen species (ROS/RNS) generation by neutrophils. Despite this, our understanding of innate immune killing and escape by *C. albicans* is primarily studied in macrophages, and the precise mechanisms of evasion are unclear in neutrophils. Here, we sought to determine the importance of neutrophil reactive nitrogen species (RNS) production during *C. albicans* infection *in vivo*. Using a zebrafish model, we found that *C. albicans* rapidly downregulated neutrophil RNS below basal levels during the first day post-infection, a time at which neutrophil RNS is upregulated in bacterial infections as an important host-defense mechanism, indicating fungal evasion of host neutrophils. We confirmed the downregulation of RNS in human primary neutrophils and with clinical *Candida* isolates, including emerging human pathogens *Candida auris* and *Candida glabrata*. Inducible nitric oxide synthase (iNOS; Nos2 in zebrafish), the enzyme responsible for RNS production, competes with the arginase enzyme for a shared substrate, L-arginine. Using a zebrafish *arginase2* transgenic line and a *C. albicans car1*Δ mutant, we showed that both host and fungal arginase contribute to the reduction in neutrophil RNS. Despite pathogen downregulation, upregulation of neutrophil RNS via hypoxia-inducible factor 1α (Hif-1α) stabilization was sufficient to improve host survival following *C. albicans* infection. Inhibition of Nos2 blocked the host protective effect of Hif-1α stabilization. Finally, restoration of neutrophil RNS via Hif-1α stabilization was additive to clinically relevant antifungal treatment, increasing survival and clearance of *C. albicans* infections. Together, these data demonstrate that restoration of the neutrophil RNS response in *C. albicans* infection improves infection outcomes, highlighting the potential of targeting Hif-1α and RNS in host-directed therapies against fungal infections.

**IMPORTANCE:**

*Candida albicans* is a fungus that normally lives harmlessly in the human body but can cause life-threatening infections in people with weakened immune systems. A key part of the body’s defense against this fungus is neutrophils, immune cells that kill microbes using toxic molecules. However, how *Candida* avoids neutrophil defense is not well understood. Here, we used zebrafish and human immune cells to show that *Candida* suppresses an important neutrophil defense, reactive nitrogen species (RNS), during infection. Unlike bacteria, which trigger RNS, *Candida* reduces these protective molecules to below normal levels, helping its survival. This effect was also observed with other disease-causing *Candida* species. We went on to show that both the host and *Candida* contribute to this suppression. Importantly, boosting the neutrophil response improved survival and helped clear infection, especially when combined with standard antifungal drugs. These findings suggest new ways to support the immune system alongside existing treatments.

## INTRODUCTION

*Candida albicans* (*C. albicans*) is a commensal fungus, colonizing 70% of healthy individuals (1) and can cause superficial mucosal infections with negligible mortality. However, *C. albicans* can act as an opportunistic pathogen capable of causing life-threatening, invasive disease in immunocompromised individuals (1–3). There are approximately 750,000 cases of severe candidiasis worldwide per year, and the estimated mortality rate is high, estimated to be around 50% (4, 5), likely an underestimation due to poor surveillance mechanisms worldwide (6, 7). This high mortality, combined with a lack of fungal vaccines and rising rates of antifungal resistance, has resulted in *C. albicans* being designated as one of four critical priority fungal pathogens by the World Health Organization, with another *Candida* species, the emerging human pathogen *Candida auris*, also a critical priority pathogen (5). Hence, urgent research into new therapies is required.

*C. albicans* is detected by pattern recognition receptors, including Toll-like receptors, C-type lectin receptors, and the Rig-I-like receptor MDA-5, resulting in a downstream pro-inflammatory response and innate immune cell recruitment (8). Although many immune cell types are recruited to the sites of *C. albicans* infection, neutrophils are one of the most potent killers of *C. albicans*, with neutropenic patients being particularly at risk of invasive disease (9, 10). Following recruitment, neutrophils have multiple mechanisms of eliminating *C. albicans*, including the production of reactive oxygen species (ROS) and reactive nitrogen species (RNS) (11). Pharmacological inhibition of nitric oxide synthase (NOS) leads to increased mortality in *C. albicans* infections in mice *in vivo* and reduced candidacidal activity in murine macrophages and peritoneal cells *in vitro*, showing the importance of RNS-mediated fungicidal activity (12–15). iNOS, the enzyme responsible for RNS production, competes with the arginase enzyme for a shared substrate, L-arginine. Arginase can competitively inhibit RNS production by iNOS in leukocytes (16). *C. albicans* is able to subvert the host innate immune response, including ROS and RNS (17). Despite *Candida* spp. being primarily controlled by neutrophils in human disease, most immune evasion studies have focused on murine macrophage cells. This is, in part, because neutrophils are a more challenging cell type to study *in vitro* due to their short lifespan and ease of accidental activation. *C. albicans* has been shown to suppress ROS in murine macrophages and neutrophils and RNS production by macrophages *in vitro* (18, 19). Culture supernatant also suppressed macrophage RNS, although to a lower extent, implying secreted compounds are partially involved in RNS suppression (19). How *Candida* spp. evade neutrophil RNS is a major knowledge gap, critical to understanding the pathogenesis of human disease. However, to overcome challenges associated with studying neutrophils *in vitro*, we require *in vivo* animal models to understand the neutrophil response in intact tissues.

Zebrafish are a highly tractable *in vivo* model system to investigate the behaviors of innate immune cells during infection (20–23). Adaptive immunity does not mature until 4–6 weeks post-fertilization, whereas innate immune cells are present from early larval stages, allowing examination of innate immune responses to infections without the presence of adaptive immunity (22). Zebrafish models of *C. albicans* infection are well-established, with a dose-dependent effect on zebrafish mortality (24), and have been used to uncover important disease mechanisms (25–27). Furthermore, *C. albicans* is able to undergo the yeast-to-hyphae transition in zebrafish larvae, demonstrating a comparable pathogenesis to human infection (24, 28–30).

Stimulation of RNS has the potential to be a host-directed therapy to *C. albicans* infection that would complement existing antifungals. We have previously shown that hypoxia-inducible factor 1α (HIF-1α) increases neutrophil RNS output, which is protective against the bacterial *Mycobacterium marinum* infection in zebrafish (31). HIF proteins are important regulators of cellular responses to hypoxia, innate immunity, and inflammation (32), triggering transcription of target genes that regulate cellular metabolism, proliferation, migration, differentiation, angiogenesis, and pro-inflammatory mediators (33–36). Therefore, we set out to address whether HIF-1α stabilization may be a potential therapeutic mechanism that can enhance neutrophil RNS during *C. albicans* infections.

Here, we show that *C. albicans* infection suppressed neutrophil RNS in zebrafish larvae, with a similar dampening effect in human neutrophils. The effect was observed with clinical strains and other *Candida* species, with the ability of strains to reduce neutrophil RNS positively correlated with virulence *in vivo*. Upregulation of host RNS production via Hif-1α stabilization enhanced host control of *C. albicans* infection, and this was additive with conventional antifungals. Together, these data highlight RNS and Hif-1α as potential targets for host-directed therapies against *C. albicans* infections.

## MATERIALS AND METHODS

### Zebrafish husbandry

Zebrafish were maintained in Home Office-approved facilities in Biological Services Unit (BSU) aquaria at the University of Sheffield, in accordance with standard protocols and local animal welfare regulations. Adult fish were maintained at 28°C with a 14/10 h light/dark cycle. Larvae were maintained at 28°C with a 14/10 h light/dark cycle in 1× E3 medium (E3 60X stock: 5.0 mM NaCl, 0.17 mM KCl, 0.33 mM CaCl_2_, 0.33 mM MgSO_4_; diluted to 1× in distilled water) with 0.001% Methylene Blue (Sigma-Aldrich) to prevent fungal growth in the first 24 h. From 24 hpf, larvae were kept in E3 without Methylene Blue so that *Candida* species infection was not impacted. The following zebrafish strains were used: nacre (wild type), *Tg(mpx:GFP)i114* (23), *Tg(lyz:nfsB.mCherry)sh260* (37), *Tg(mpeg:nlsClover*)sh436 (38), *TgBAC(arg2:GFP)sh571* (39), and *Tg(phd3:GFP)i144* (40).

### *Candida* species culture

*C. albicans* strains (Table 1) and *Candida* species clinical isolates (Table 2) were used in this study. *C. albicans* was initially grown on YPD (4001022, MP Bio) plates at 28°C for 48 h and then transferred to overnight liquid culture in YPD broth in a shaking incubator at 30°C at 200 rpm. *C. albicans* was heat-killed by incubation in a heat block at 65°C for 1 h (41). To denature fungal proteins, *C. albicans* was boiled at 100°C for 5 min.

**TABLE 1:** List of *C. albicans* strains

Strain	Genotype	Reference
*C. albicans* TT21-dTomato	*ade2::hisG/ade2::hisG ura3::imm434/ura3::imm434::URA3- tetO ENO1/eno1*::ENO1 tetR-ScHAP4AD-3XHA-ADE2 pENO1-dTomato-NATR	(29)
*C. albicans* SN148 GFP	SN148 *ENO1/ENO1-GFP::LEU2*	(30)
*C. albicans* SC5314	wt; clinical blood isolate	(42)
*C. albicans car1*Δ	As SC5314 but *car1*Δ*car1*Δ	(43)
*C. albicans NRG1^OEX^-dTomato*	e2::hisG/ade2::hisG *ura3::imm434/ura3::imm434::URA3-tetO-NRG1 ENO1/eno1*::ENO1 tetR-ScHAP4AD-3XHA-ADE2 pENO1-dTomato-NATR	(29)
*C. albicans apm4*Δ/Δ	As SN148 but *apm4*Δ*apm4*Δ	(30, 44)

**TABLE 2:** List of *Candida* species clinical isolates

Species	Name	From invasive or non-invasive disease	Sample site	Source
*Candida albicans*	AJP4	Non-invasive	Vaginal sample	Sheffield Teaching Hospital’s Trust
*Candida albicans*	AJP5	Invasive	Blood sample	Sheffield Teaching Hospital’s Trust
*Candida albicans*	AJP9	Non-invasive	Tongue swab	Sheffield Teaching Hospital’s Trust
*Candida albicans*	AJP25	Invasive	Line tip sample	Sheffield Teaching Hospital’s Trust
*Candida glabrata*	AJP12	Invasive	Urine sample	Sheffield Teaching Hospital’s Trust
*Candida parapsilosis*	AJP22	Invasive	Urine sample	Sheffield Teaching Hospital’s Trust
*Candida guilliermondii*	AJP24	Non-invasive	Tracheal swab	Sheffield Teaching Hospital’s Trust
*Candida auris*	StG2	Invasive	Bloodstream	City St George’s University of London

### Zebrafish infection

Overnight liquid *C. albicans* cultures were washed three times in phosphate-buffered saline (PBS) and counted on a hemocytometer. Washed cultures were resuspended in 10% polyvinylpyrrolidone (PVP; Calbiochem) to achieve the desired inoculation. Infection dose for systemic caudal vein infections was 200 colony-forming units (cfu) in a 1 nL injection volume, 100 cfu in 1 nL for experiments with immune cell ablation, or 500 cfu in 1 nL in experiments with antifungals. Dosing was performed to achieve around 50% of zebrafish death by 4 days post-infection (dpi; equivalent to 5 days post-fertilization, dpf) (Fig. S1A available at https://doi.org/10.15131/shef.data.32630970), and injected cfu were validated by plating (Fig. S1B available at https://doi.org/10.15131/shef.data.32630970).

For survival curves, zebrafish survival was monitored daily, and dead zebrafish were removed. Death was determined by a cessation of heartbeat/circulation or tissue degradation post-mortem. At 4 dpi, all surviving zebrafish larvae were culled.

For *Staphylococcus aureus* experiments, 100 cfu of strain SH1000 was injected into the caudal vein in a 1 nL injection volume, as previously described (45).

### RNA injections

Larvae were injected with dominant active *hif-1*α*b* variant RNA (DA *hif-1*α) or dominant-negative *hif-1*α*b* variant (DN *hif-1*α) RNA in dH_2_O with 10% phenol red (Sigma-Aldrich, to visualize successful injection; 1 nL total injection volume) at the one-cell stage, as previously described (46); 10% (vol/vol) phenol red in dH_2_O (PR; Sigma-Aldrich) was used as a solvent control.

### Morpholino injections

A *csf3r* morpholino (Gene Tools) was used to deplete neutrophil populations (47). A standard control morpholino (Gene Tools) was used as a negative control. The antisense morpholino oligonucleotide sequences were as follows: *csf3r*, 5′-GAAGCACAAGCGAGACGGATGCCAT-3′, and control, 5′-CCTCTTACCTCAGTTACAATTTATA-3′. *csf3r* morpholino was functionally validated by whole-body neutrophil count at 2 dpf.

### F0 CRISPR-Cas9 knockdown

Two sgRNAs were designed to target the ATG/first exon of *nos2a* and *nos2b* to knock down zebrafish *nos2* genes by CRISPR-Cas9. Efficacy of knockdown of RNS production was functionally validated by whole-body anti-nitrotyrosine staining. Tyrosinase (*tyr*) sgRNA, targeting a pigment gene that plays no role in neutrophil RNS production, was used as a negative control and to demonstrate Cas9 function (48). The target sequences were as follows: *nos2a*, 5′-TTTCTCATTTTCAATGATAG-3′; *nos2b*, 5′-GTTCGCTCTTGTGAGTGACC-3′; and *tyr*, 5′-CCTGACCTCCTGAAGACCCC-3′.

In total, 25 μM sgRNA was co-injected with tracrRNA, Cas9 protein, and phenol red in a total injection volume of 1 nL.

### Pharmacological treatment of zebrafish larvae

For pharmacological inhibition of nitric oxide synthase, larvae were treated with 200 μM N6-(1-iminoethyl)-lysine (L-NIL) by immersion. dH_2_O was used as a solvent control (31). For pharmacological stabilization of Hif-α, larvae were treated with 5.0 μM FG4592 (Roxadustat, Selleckchem) by immersion. DMSO was used as a solvent control (49).

For antifungal survival curves and clearance assays, larvae were treated with either 5.0 µg/mL fluconazole in DMSO or 1.0 µg/mL caspofungin in dH_2_O by immersion. DMSO or dH_2_O was used as a solvent control. At 2 dpi, larvae were transferred into fresh E3, and treatment was re-administered.

### Anti-nitrotyrosine staining

One day post-infection, larvae (2 dpf) were fixed in 4% formaldehyde in PBS overnight at 4°C. Whole-body nitrotyrosine levels were labeled using a rabbit polyclonal anti-nitrotyrosine antibody (06-284; Merck Millipore) and were detected using Alexa-633 conjugated goat-anti-rabbit secondary antibody (Invitrogen Life Technologies), as previously described (31, 50) in a T*g(mpx:GFP)i114* background to assess for neutrophil-specific RNS.

### Microscopy and quantification of anti-nitrotyrosine staining

For *C. albicans* SC5314 and *car1*Δ, brightfield images were taken using an inverted Leica DMi8 with a 40× lens and a Hamamatsu OrcaV4 camera. Stained larvae were imaged on a Leica DMi8 inverted microscope with a Leica TCS-SPE line-scanning confocal for imaging with a 40 × 1.1 NA water immersion lens. For quantification purposes, acquisition settings were kept the same across the groups. Corrected fluorescence intensity was calculated using FIJI measurements assessing the cell fluorescence of individual neutrophils corrected for cell size and background fluorescence of the image (31, 50, 51).

### Hyphal staging

For assessment of fungal growth of *Candida* species clinical isolates, which lack fluorescent proteins, brightfield images were taken using an inverted Leica DMi8 with a 40x lens and a Hamamatsu OrcaV4 camera. Classification of hyphal stages was done manually, with the experimenter blinded to experimental groups, based on a previously described descriptive criteria (52). Criteria for hyphal staging were as follows.

1. Yeast: only spherical, yeast cells were observed. No signs of any hyphal growth.
2. Germinating: ovoid cells, resembling pseudohyphae or very short hyphae, were observed.
3. Hyphal extension: a small number of short- or medium-length hyphae were observed.
4. Destructive hyphae: multiple invasive hyphae were observed. The majority of observed *C. albicans* was in hyphal morphotype.

### Human neutrophils

Human neutrophils were isolated by Plasma-Percoll density gradient centrifugation of whole blood from healthy donors as previously described (53); 0.5 × 10^6^ cells were added to individual wells in 96-well plates in 100 μL volumes. Cells were primed with 1 μg/mL lipopolysaccharides (LPS) from *Escherichia coli* (Sigma, L8274). Neutrophils were infected with *Candida albicans* at an MOI of 1.0 or 0.5, with heat-killed *C. albicans* at an MOI of or 1.0, left uninfected (sterile RPMI media), and were incubated at 37°C, 5% CO_2_ for 3 h. Cells were cytocentrifuged onto microscope slides and fixed in 4% formaldehyde for staining. Immunostaining was performed as described above.

### Microscopy of *C. albicans* infection clearance

Brightfield images were taken using an inverted Leica DMi8 with a 2.5× lens and a Hamamatsu OrcaV4 camera. Surviving fish were classified as “infection cleared” only if no signs of remaining fluorescent *C. albicans* infection could be observed at 2.5× magnification.

### Statistical analysis

All data were analyzed using GraphPad Prism 10.5.0 1 (GraphPad Software, La Jolla, CA, USA, https://www.graphpad.com/). Nitrotyrosine staining data were analyzed with a two-tailed Mann-Whitney test or Kruskal-Wallis test, with Dunn’s multiple comparisons test. Survival curves were analyzed with the Gehan-Breslow-Wilcoxon test, with Bonferroni correction. The *P* values shown are: ns = not significant, *P < 0.05, **P < 0.01, ***P < 0.001, ****P < 0.0001

## RESULTS

### *C. albicans* infection suppresses neutrophil RNS production below basal levels

**Fig 1:**
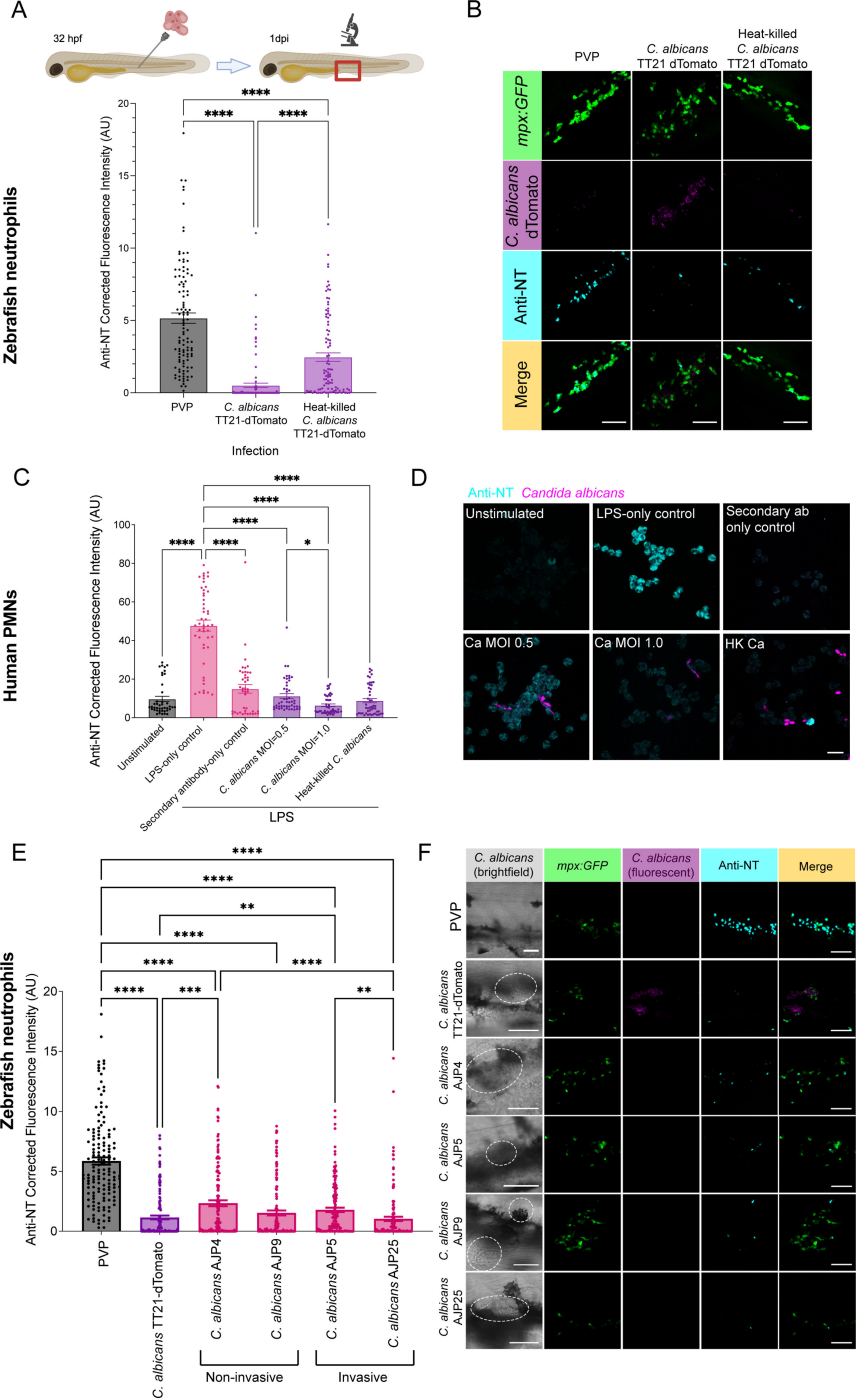
*C. albicans* suppressed neutrophil RNS production below resting levels. (**A**) Schematic of experiment: 1 dpf zebrafish were injected with PVP or *C. albicans* into the caudal vein. At 1 dpi, larvae were fixed and stained with anti-nitrotyrosine primary antibody and goat anti-rabbit Alexa-633 secondary antibody. Zebrafish were then imaged at the site of infection in the caudal hematopoietic tissue (CHT), and anti-nitrotyrosine fluorescence was quantified. The graph shows anti-nitrotyrosine fluorescence at 1 dpi following injection of PVP or 200 cfu of live *C. albicans* TT21-dTomato or heat-killed *C. albicans* TT21-dTomato into the caudal vein at 30 hpf. *N* = 96–108 neutrophils from 16 to 18 fish, obtained from three independent experiments. Error bars show SEM. Statistical significance determined by Kruskal-Wallis test, and then Dunn’s multiple comparisons test. *P* values are shown: ****P < 0.0001. (**B**) Representative images of PVP, *C. albicans* TT21-dTomato, and heat-killed *C. albicans* TT21-dTomato-infected zebrafish larvae at 1 dpi. Scale bars = 50 µm. (**C**) Corrected fluorescence intensity of PMNs fixed at 3 h post-LPS treatment and stained with anti-nitrotyrosine antibody (in arbitrary units [AU]). Non-LPS-stimulated resting neutrophils were used as unstimulated controls (gray bar). LPS-stimulated neutrophils were included as controls, either stained with primary (anti-NT antibody) and secondary antibody (goat-anti-rabbit Alexa-633) (LPS-only control), or secondary alone (Secondary antibody-only control) (pink bars). Neutrophils infected with an MOI of 0.5 and 1.0 live *C. albicans* and heat-killed *C. albicans* all show a decrease in nitrotyrosine in the presence of LPS compared to LPS-only controls (purple bars). *N* = 42–48 neutrophils from two independent experiments and donors. (**D**) Representative images of human PMNs stained with anti-nitrotyrosine (anti-NT). Scale bars = 50 µm. *P < 0.05, ****P < 0.0001. (**E**) Anti-nitrotyrosine fluorescence at 1 dpi following injection of PVP, *C. albicans* TT21-dTomato, or *C. albicans* clinical isolates into the caudal vein at 30 hpf. *N* = 144 neutrophils from 24 fish, obtained from three independent experiments. Error bars show SEM. Statistical significance determined by Kruskal-Wallis test and then Dunn’s multiple comparisons test. *P* values shown: **P < 0.01, ***P < 0.001, ****P < 0.0001. (**F**) Representative images of PVP, *C. albicans* TT21-dTomato, *C. albicans* AJP4, *C. albicans* AJP5, *C. albicans* AJP9, and *C. albicans* AJP25 infected zebrafish larvae at 1 dpi. White dotted circles show *C. albicans*. Scale bars = 50 µm.

Live *C. albicans* caused a 90.0% reduction in the levels of RNS (visualized using an anti-nitrotyrosine antibody) compared to mock infection (PVP) controls (Fig. 1A and B), indicating not only a lack of induction of RNS, but also a downregulation of basal RNS. We have previously shown that RNS is predominantly found in neutrophils both in unchallenged and mock-infected (PVP) individuals (50) and is upregulated after bacterial challenge with *Mycobacterium marinum* (31). This established that *C. albicans* suppressed neutrophil RNS *in vivo* in contrast to bacterial challenge. In mock-infected (PVP) controls, there was a basal level of nitrotyrosine in neutrophils, as observed previously (31, 50). We tested another bacterium, *Staphylococcus aureus,* to determine if induction of neutrophil RNS was limited to mycobacteria. *S. aureus* (SH1000) infection also caused a significant increase in neutrophil nitrotyrosine fluorescence (Fig. S2 available at https://doi.org/10.15131/shef.data.32630970), corroborating previous observations of increased RNS levels in response to mycobacterial infection (31). RNS reduction by *C. albicans* was not limited to a single strain, as we infected with a different *C. albicans* strain, SN148 GFP, and observed similar RNS suppression (Fig. S3 available at https://doi.org/10.15131/shef.data.32630970). This allowed the use of TT21-dTomato and SN148 GFP interchangeably, depending on the color combinations required for each experiment. Zebrafish larvae infected with heat-killed C. albicans TT21-dTomato had an intermediate level of RNS suppression, between the PVP control and live C. albicans infection (Fig. 1A and B). These data indicate that the full effect of neutrophil RNS suppression is dependent on live *C. albicans* but can be suppressed to a lesser extent by heat-killed *C. albicans*, suggestive of a combination of active and passive mechanisms.

To confirm our finding in humans, we performed anti-nitrotyrosine staining on human primary polymorphonuclear neutrophils (PMNs) after *C. albicans* infection *in vitro*. Detectable and robust RNS induction in human neutrophils was induced by priming with LPS, which led to a robust nitrotyrosine response after 3 h in the absence of infection (LPS mock-infected) (Fig. 1C and D). When *C. albicans* was added at an MOI of 1.0, nitrotyrosine was suppressed to baseline unstimulated levels, despite the presence of LPS (Fig. 1C and D). At a lower MOI of 0.5, live *C. albicans* showed less RNS suppression compared to an MOI of 1.0 of live *C. albicans*, indicating a dose-dependent effect (Fig. 1C and D). Similarly, an MOI of 1.0 of heat-killed *C. albicans* exhibited lower RNS suppression compared to the same MOI of live *C. albicans*. Together, these data show that *C. albicans* suppresses RNS production in human neutrophils as in zebrafish.

The laboratory reference strain SC5314 and strains derived from this (including SN148 and TT21) have inactivating missense mutations in RNAi component Argonaute, and hence, they may behave differently from *C. albicans* strains found in the clinic (54). Therefore, clinical isolates from patients with invasive (AJP5 and AJP25) and non-invasive (AJP4 and AJP9) disease were tested for neutrophil RNS suppression in zebrafish. Our clinical isolates lacked a fluorescent protein marker; hence, brightfield microscopy was used to identify sites of infection in anti-nitrotyrosine stained zebrafish larvae. Isolates from both invasive (AJP5 and AJP25) and non-invasive (AJP4 and AJP9) disease in patients were able to significantly reduce zebrafish neutrophil RNS compared to mock infection (PVP) controls (Fig. 1E and F). These observations demonstrated that neutrophil RNS suppression by *C. albicans* is conserved in clinical isolates, implying that RNS suppression in *C. albicans* infections may be a clinically relevant phenomenon.

### Full neutrophil RNS suppression is partially dependent on *C. albicans* hyphae formation

**Fig 2:**
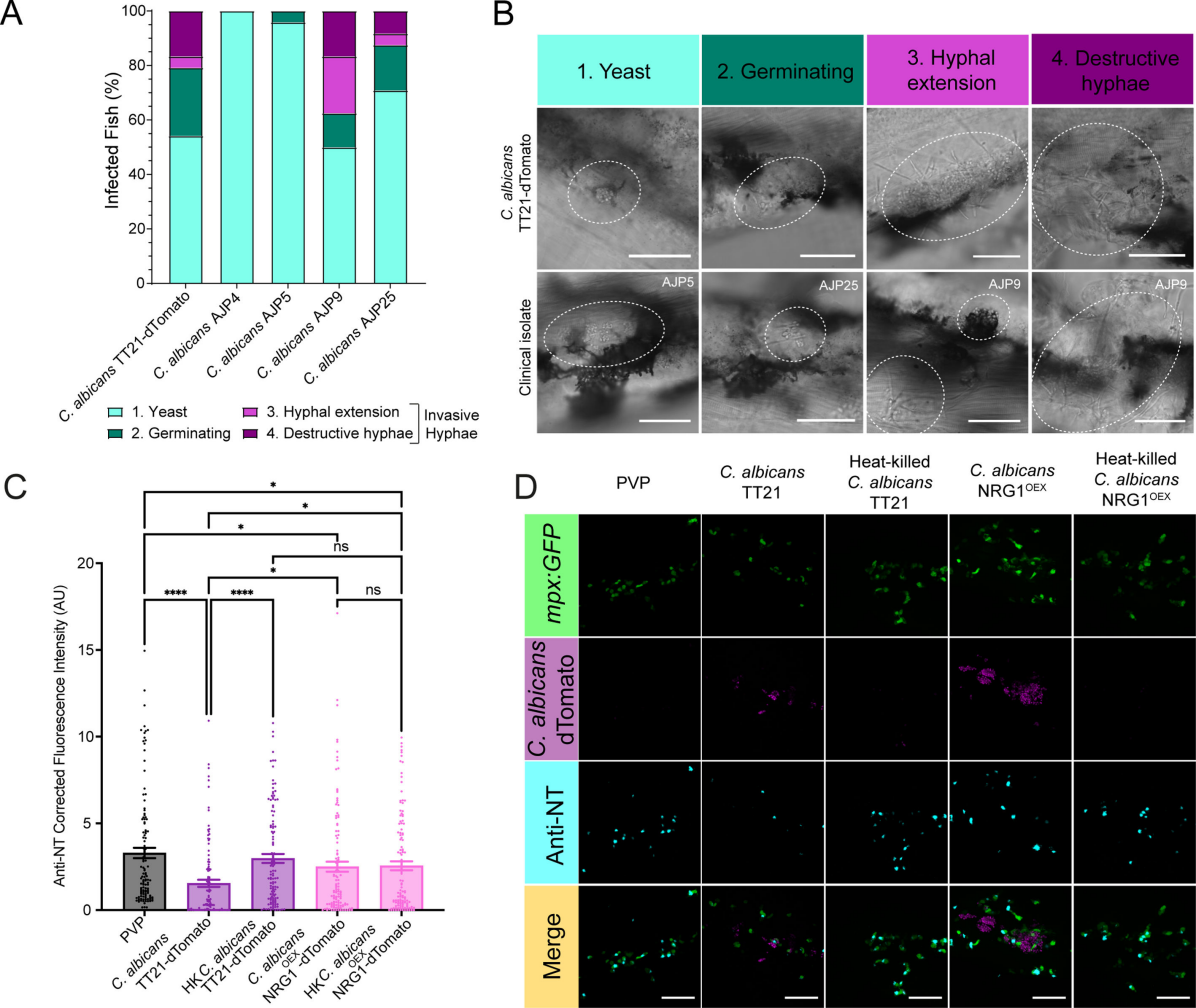
Robust RNS suppression was associated with hypha-forming *C. albicans.* (**A**) Hyphal classification of zebrafish larvae infected with a range of *C. albicans* isolates at 1 dpi. *N* = 24 fish, obtained from three independent experiments. (**B**) Representative images of hyphal classification in 1 dpi zebrafish larvae infected with *C. albicans* TT21-dTomato or a *C. albicans* clinical isolate. Images are labeled with the *C. albicans* isolate. White-dotted circles show regions of fungal growth. Scale bar = 50 µm. (**C**) Anti-nitrotyrosine fluorescence at 1 dpi following injection of PVP, *C. albicans* TT21-dTomato, heat-killed *C. albicans* TT21-dTomato*, C. albicans* NRG1^OEX^-dTomato, or heat-killed *C. albicans* NRG1^OEX^-dTomato into the caudal vein. *N* = 120 neutrophils from 20 fish, obtained from three independent experiments. Error bars show SEM. Statistical significance was determined by Kruskal-Wallis test, with Dunn’s multiple comparisons tests. *P < 0.05, ****P < 0.0001. (**D**) Representative images of PVP, *C. albicans* TT21-dTomato, heat-killed *C. albicans* TT21-dTomato*, C. albicans* NRG1^OEX^-dTomato or heat-killed *C. albicans* NRG1^OEX^-dTomato infected larvae at 1 dpi. Scale bars = 50 µm.

*C. albicans* morphological switch from yeast to hyphae is associated with increased virulence and shifts in the transcriptome and secretome (55–57). Classification of hyphal growth by *C. albicans* TT21 and clinical isolates in zebrafish revealed that the isolates associated with greater neutrophil RNS suppression (AJP9 and AJP25) (Fig. 1E and F) also exhibited greater hyphal growth *in vivo* compared to those that reduced RNS to a lesser extent (AJP4 and AJP5) (Fig. 2A and B). Based on this correlation, we hypothesized that hyphal switching may be partially responsible for the suppression of neutrophil RNS. To investigate this, 1 dpf zebrafish were infected with *C. albicans* TT21-dTomato or yeast-locked strain *C. albicans* NRG1^OEX^-dTomato (29). Both *C. albicans* NRG1^OEX^-dTomato and heat-killed *C. albicans* NRG1^OEX^-dTomato caused a modest decrease in RNS compared to wild-type strains, with reductions comparable to heat-killed *C. albicans* (Fig. 2C and D). These data suggest that full neutrophil RNS suppression by *C. albicans* is partially hyphal switch-dependent.

During hypha formation, synthesis of cell wall components, such as chitin, is upregulated (58). To determine whether *C. albicans* chitin is involved in RNS suppression, a strain lacking AP-2 (*apm4*Δ/Δ), which has higher chitin levels but decreased virulence in zebrafish due to defective hyphae formation (30), was investigated. Despite defects in hypha formation, *apm4*Δ/Δ suppressed RNS to similar levels as wild-type *C. albicans*, suggestive of a role for the cell wall component chitin in RNS suppression (Fig. S4 available at https://doi.org/10.15131/shef.data.32630970).

### Host arginase is induced by *C. albicans*

**Fig 3:**
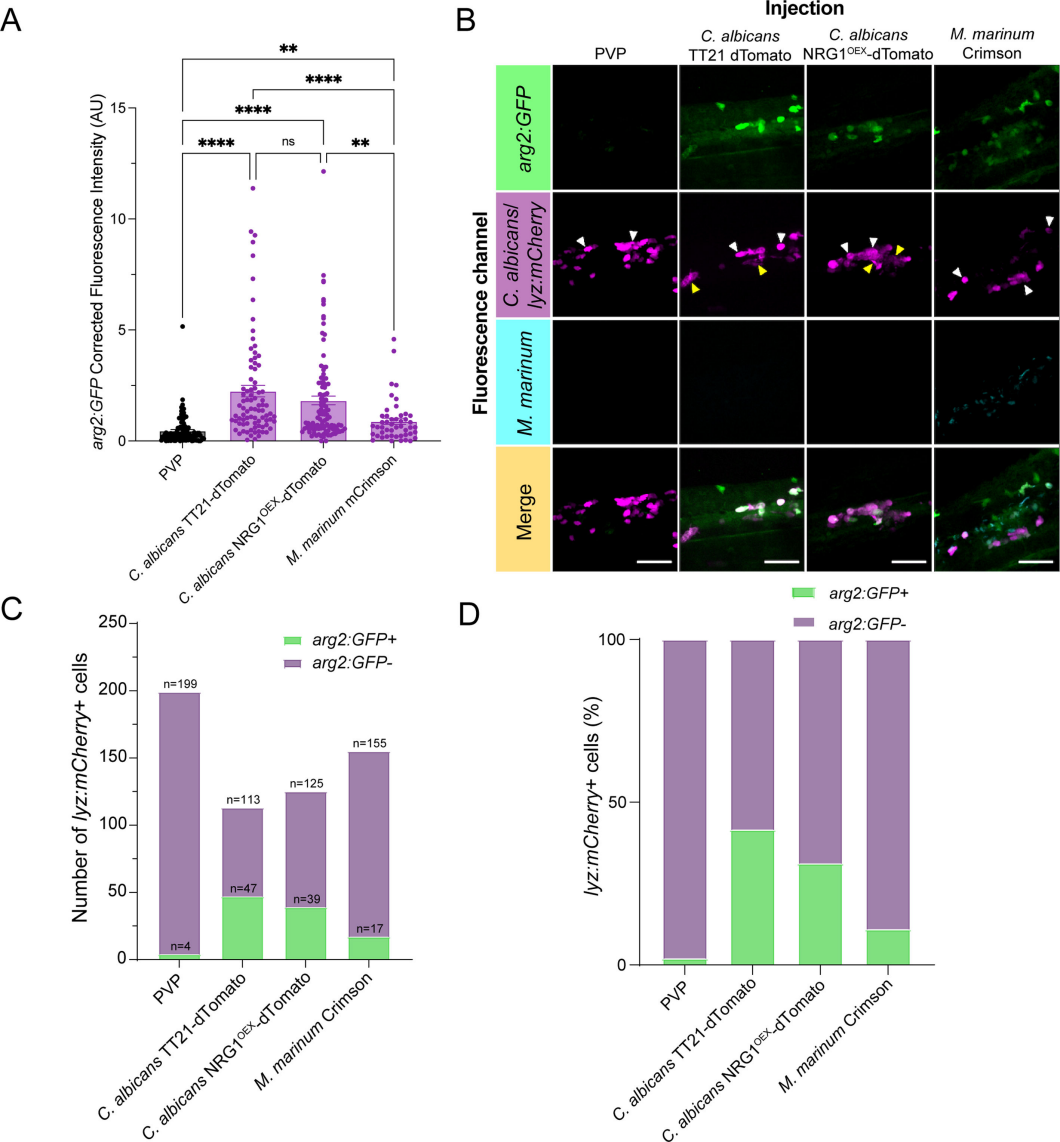
*C. albicans* infection stimulated host *arginase* 2 expression. (**A**) Corrected fluorescence intensity of *Tg(arg2:GFP*) zebrafish larvae at 1 dpi following injection of PVP, *C. albicans* TT21-dTomato, *C. albicans* NRG1^OEX^-dTomato, or *M. marinum* Crimson into the caudal vein at 30 hpf. For PVP, *C. albicans* TT21-dTomato, and *C. albicans* NRG1^OEX^-dTomato: *n* = 84–108 cells from 14 to 18 fish, obtained from three independent experiments. For *M. marinum*: *n* = 48 cells from eight fish, obtained from two independent experiments. Error bars show SEM. Statistical significance determined by Kruskal-Wallis test, then Dunn’s multiple comparisons test. *P* values shown: **P < 0.01, ****P < 0.0001. (**B**) Representative images of *Tg(arg2:GFP*) zebrafish embryos injected with PVP, *C. albicans* TT21-dTomato, *C. albicans* NRG1^OEX^-dTomato, or *M. marinum* crimson at 24 hpi. White arrowheads point toward neutrophils. Yellow arrowheads point toward *C. albicans*. Scale bars = 50 µm. (**C**) Number of *lyz:nfsB.mCherry*-expressing cells that are *arg2:GFP*+ and *arg2:GFP−,* following infection with *C. albicans* TT21-dTomato, *C. albicans* NRG1^OEX^-dTomato, *M. marinum* Crimson or PVP. *N* = 113–199 cells from 12 zebrafish, obtained from two independent experiments. (**D**) Percentage of *lyz:nfsB.mCherry*-expressing neutrophils that are *arg2:GFP*+ and *arg2:GFP−,* following infection with *C. albicans* TT21-dTomato, *C. albicans* NRG1^OEX^-dTomato, *M. marinum* Crimson, or PVP. *N* = 113–199 cells from 12 zebrafish, obtained from two independent experiments.

**Fig 4:**
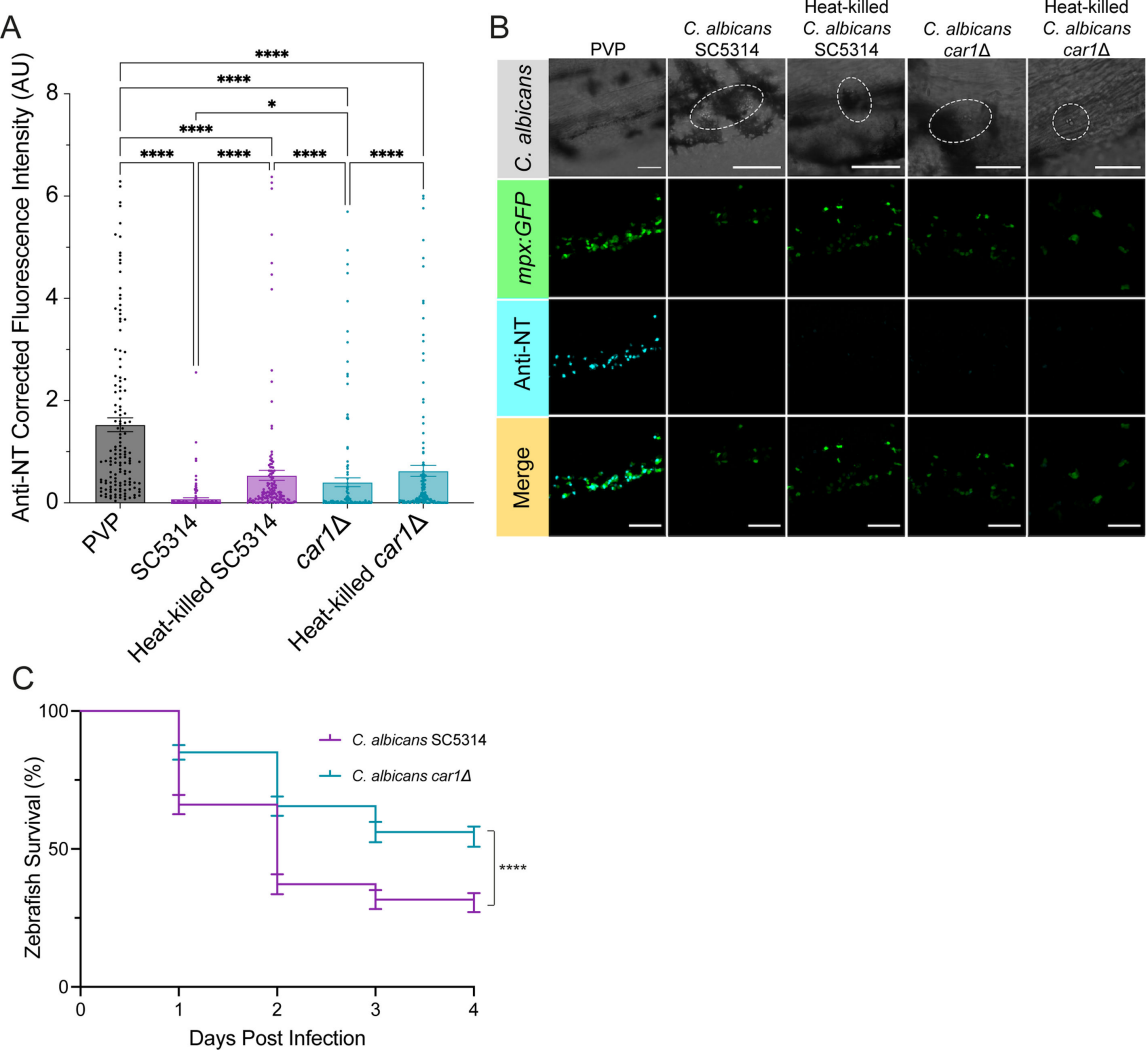
*C. albicans car1* contributed to suppression of neutrophil RNS. (**A**) Anti-nitrotyrosine fluorescence at 1 dpi following injection of PVP, *C. albicans* SC5314*,* heat-killed *C. albicans* SC5314*, C. albicans car1*Δ, or heat-killed *C. albicans car1*Δ into the caudal vein. *N* = 144 neutrophils from 24 fish, obtained from three independent experiments. Error bars show SEM. Statistical significance determined by Kruskal-Wallis test, with Dunn’s multiple comparisons tests. *P < 0.05, ****P < 0.0001. (**B**) Representative images of anti-nitrotyrosine-stained larvae. Dashed lines show *C. albicans*. Scale bars = 50 µm. (**C**) One dpf nacre larvae were injected into the caudal vein with 200 cfu *C. albicans* SC5314 or *C. albicans car1*Δ. Larval survival was measured daily up to 4 dpi. *N* = 180 fish, obtained from three independent experiments. Error bars show SEM. Statistical significance determined by Gehan-Breslow-Wilcoxon test, with Bonferroni correction. *P* values shown: ****P < 0.0001

Inducible nitric oxide synthase (iNOS; Nos2 in zebrafish) is the enzyme responsible for RNS production. iNOS competes with the arginase enzyme for a shared substrate, L-arginine, and arginase can competitively inhibit RNS production by iNOS (16). We investigated the impact of *C. albicans* infection on host arginase levels. PVP-injected larvae had a low basal level of *arg2:GFP* expression (Fig. 3A and B). *Mycobacterium marinum* (used as a positive control for arginase induction) caused a significant upregulation of *arg2:GFP* compared to PVP, as previously described (39). *C. albicans* TT21-dTomato caused a greater upregulation in *arg2:GFP* expression compared to *M. marinum,* indicating a robust host arginase upregulation. *C. albicans* NRG1^OEX^-dTomato also caused upregulation of *arg2:GFP* compared to both PVP and *M. marinum,* indicating that this is a hyphal-independent process. Examination of *arg2:GFP* expression in neutrophils (labeled by *mpx:GFP*) revealed *C. albicans* TT21-dTomato caused an increase in the percentage of *arg2:GFP +* neutrophils (41.59% of the neutrophil population in the field of view; Fig. 4C and D) compared to PVP (2.01%) and *M. marinum* (10.97%). Together, these results reveal *C. albicans* infection upregulates host arginase expression in a subpopulation of neutrophils to a greater level than the professional bacterial pathogen *M. marinum*.

### *C. albicans*-derived arginase contributes to the suppression of neutrophil RNS

*C. albicans* produces its own version of arginase, *CAR1* (43). We hypothesized that *C. albicans* arginase interfered with the host RNS production, resulting in neutrophil RNS suppression; 1 dpf zebrafish were infected with *C. albicans car1*Δ or its parental strain *C. albicans* SC5314, and RNS production was measured at 1 dpi by anti-nitrotyrosine staining. *C. albicans car1*Δ infection decreased neutrophil RNS production compared to PVP (Fig. 4A and B). *C. albicans car1*Δ caused an intermediate level of RNS suppression between live *C. albicans* SC5314 and heat-killed *C. albicans* SC5314 (Fig. 4A and B), indicating that CAR1 contributes to the suppression of neutrophil RNS. Heat-killed *C. albicans car1*Δ was not significantly different from heat-killed *C. albicans* SC5314. Infection with *C. albicans car1*Δ resulted in significantly greater zebrafish larvae survival than infection with *C. albicans* SC5314 (Fig. 4C), suggesting that RNS suppression by car1 is important for *C. albicans* virulence *in vivo*.

### The level of neutrophil RNS suppression by *Candida* spp. correlates with virulence

**Fig 5:**
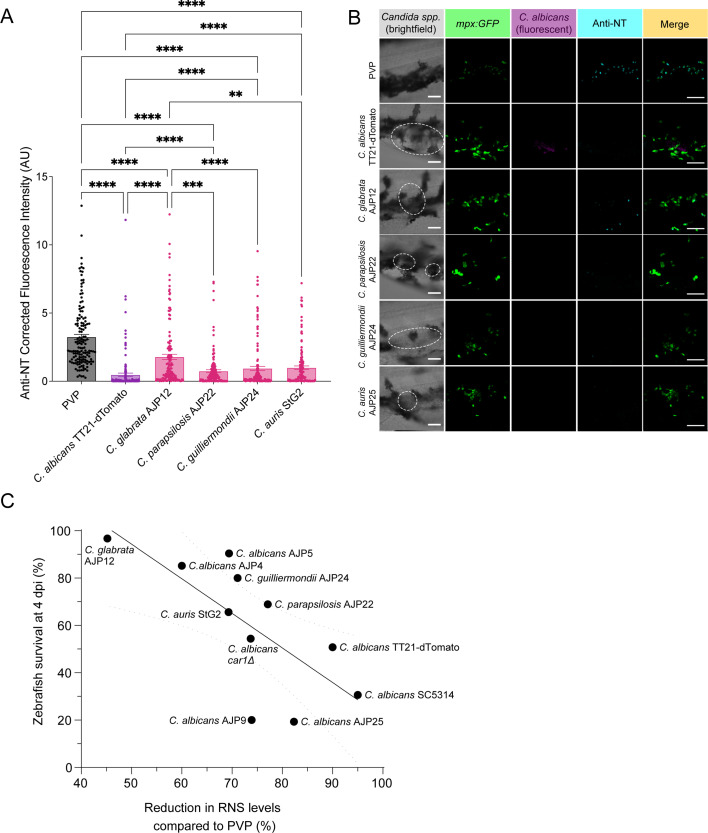
Non-albicans *Candida* spp. suppressed neutrophil RNS. (**A**) Anti-nitrotyrosine fluorescence at 1 dpi following injection of PVP, *C. albicans* TT21-dTomato, *C. glabrata* AJP12, *C. parapsilosis* AJP22, *C. guilliermondii* AJP24, or *C. auris* StG2 into the caudal vein. *N* = 144 neutrophils from 24 fish, obtained from three independent experiments. Error bars show SEM. Statistical significance determined by Kruskal-Wallis test, with Dunn’s multiple comparisons tests. * indicates difference compared to PVP. † indicates difference compared to *C. albicans* TT21-dTomato. ‡ indicates difference compared to *C. glabrata* AJP12. *P* values shown: **P < 0.01, ***P < 0.001, ****P < 0.0001. (**B**) Representative images of PVP, *C. albicans* TT21-dTomato, *C. glabrata* AJP12, *C. parapsilosis* AJP22, *C. guilliermondii* AJP24, or *C. auris* StG2 infected larvae at 1 dpi. Scale bars = 50 µm. (**C**) Reduction in RNS levels compared to PVP control plotted against zebrafish survival at 4 dpi, following systemic infection with 200 cfu *Candida* spp. Full survival curves for *Candida* spp. are in . Each plotted point is based on three independent repeats of the relevant experiment. Line of best fit and 95% confidence error lines are shown. Pearson’s correlation coefficient and *R*^2^ value were calculated.

*C. albicans* is the leading causative agent of candidiasis, but there are many other clinically important *Candida* spp. We investigated the effect of a range of *Candida* species clinical isolates on neutrophil RNS levels *in vivo*. Clinical isolates of *C. parapsilosis* (AJP22), *C. guilliermondii* (AJP24), and *C. auris* (StG2) all robustly reduced neutrophil RNS compared to PVP controls, but to a lesser extent than a laboratory strain of *C. albicans* (Fig. 5A and B). *C. glabrata* AJP12 caused a more modest decrease in neutrophil RNS compared to *C. albicans*. (Fig. 5A and B). Together, these results show that neutrophil RNS suppression is conserved across *Candida* species clinical isolates but that levels of RNS suppression can vary between species.

Intriguingly, when percentage zebrafish survival at 4 dpi was plotted against the percentage reduction of RNS levels by *C. albicans* strains/isolates compared to PVP control, survival negatively correlated with reduction in neutrophil RNS levels (Fig. 5C. Full survival curves for *Candida* spp. are in Fig. S5 and S6 (available at https://doi.org/10.15131/shef.data.32630970) (Pearson’s correlation coefficient: −0.718, 95% CI: −0.208 to −0.921; *P* < 0.05, *R*^2^ value: 0.516). Therefore, a greater suppression of host RNS levels is correlated with increased virulence in zebrafish infection *in vivo*, indicating that suppression of neutrophil RNS is a potential virulence mechanism in human disease-relevant *Candida* spp.

### Increased neutrophil RNS, via Hif-1α stabilization, is protective against *C. albicans* infection

Hif-1α is a transcription factor with known roles in regulation of innate immunity (32). We have previously shown that stabilized Hif-1α (using injection of a dominant active variant RNA) is protective against *M. marinum* infection *in vivo* by increasing neutrophil RNS levels (31, 36). We therefore hypothesized that stabilization of Hif-1α would restore neutrophil RNS in *C. albicans* infection and increase host survival.

**Fig 6:**
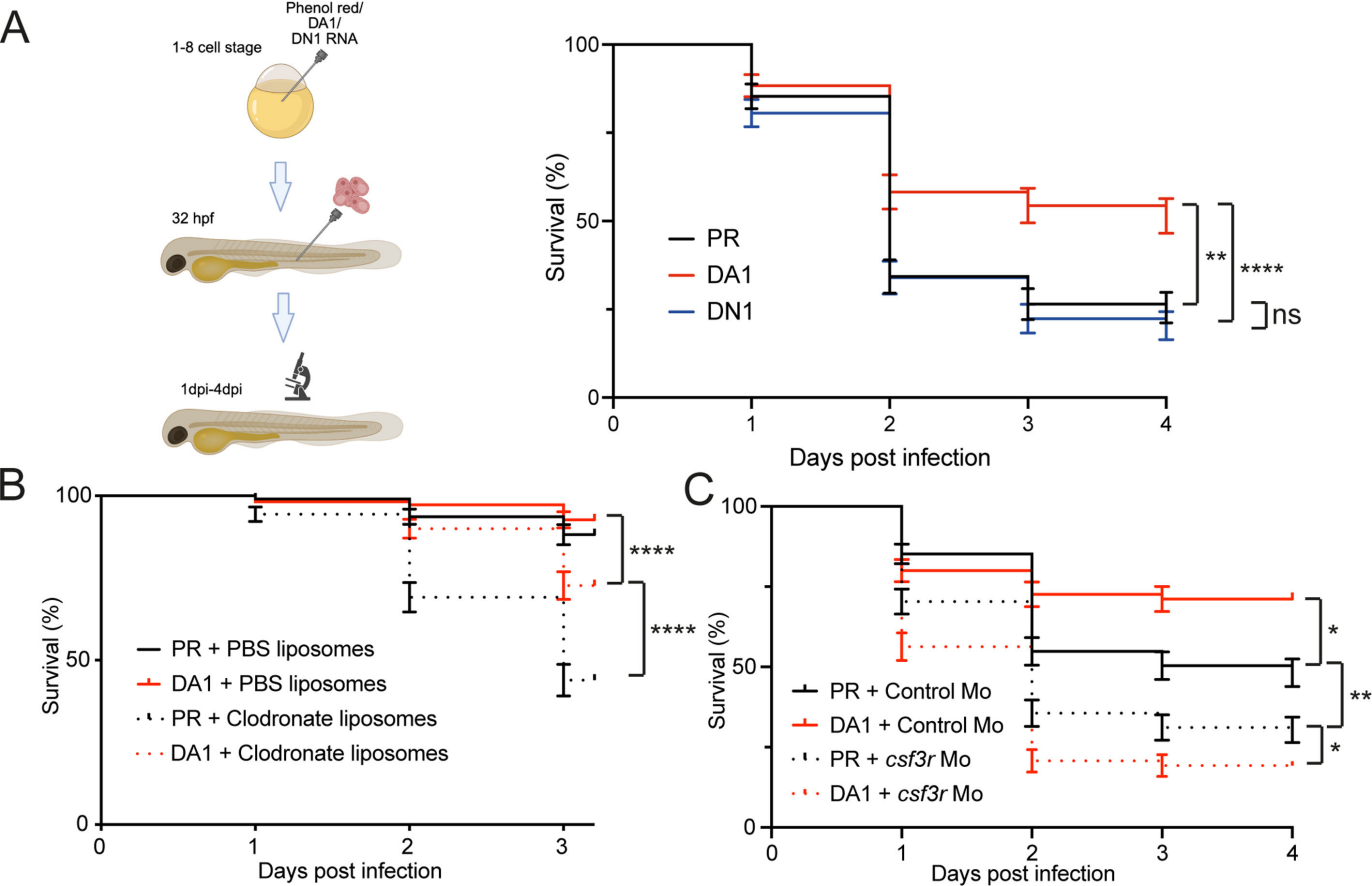
Hif-1α stabilization was protective against *C. albicans* infection. (**A**) Survival of *C. albicans* TT21-dTomato-infected zebrafish larvae following injection with DA1, DN1 RNA, or phenol red (PR) control. Mortality was measured daily. *N* = 102–103 fish, obtained from three independent experiments. Error bars show SEM. Statistical significance determined by the Gehan-Breslow-Wilcoxon test with Bonferroni correction. **P < 0.01, ****P < 0.0001. (**B**) Survival curve of 100 cfu *C. albicans* TT21-dTomato-infected zebrafish larvae following injection with DA1 or PR, then treatment with PBS or clodronate liposomes. Mortality was measured daily. *N* = 102–103 fish, obtained from three independent experiments. Error bars show SEM. Statistical significance determined by the Gehan-Breslow-Wilcoxon test with Bonferroni correction. ****P < 0.0001. (**C**) Survival curve of 100 cfu *C. albicans* TT21-dTomato-infected zebrafish larvae following injection with PR or DA1 and control or *csf3r* morpholino. Mortality was measured daily. *N* = 135 fish, obtained from three independent experiments. Error bars show SEM. Statistical significance determined by the Gehan-Breslow-Wilcoxon test with Bonferroni correction. *P < 0.05, **P < 0.01.

One-cell-stage zebrafish larvae were injected with water/10% phenol red (solvent) control (PR), dominant-negative *hif-1*α (DN *hif-1*α), or dominant-active *hif-1*α (DA *hif-1*α) RNA (as previously described (46)) and subsequently infected systemically with *C. albicans* TT21-dTomato infection at 30 hpf. DA *hif-1*α larvae had significantly greater survival over the first 4 days post-infection than both PR and DN *hif-1*α larvae (Fig. 6A), demonstrating that Hif-1α stabilization is host-protective in *C. albicans* infection. *C. albicans* infection was not sufficient to upregulate the *Tg(phd3:GFP)i144* Hif reporter transgenic line (40), which may indicate why DN *hif-1*α larvae did not affect zebrafish survival post*-C. albicans* infection (Fig. S7 available at https://doi.org/10.15131/shef.data.32630970).

Neutrophils play an essential role in innate immune control of invasive candidiasis (59). In zebrafish, neutrophils are responsible for the killing of *C. albicans*, whereas macrophages internalize but do not kill (60). Therefore, we hypothesized that the protective effect of Hif-1α stabilization would be mediated by neutrophils rather than macrophages. First, to assess the impact of Hif-1α stabilization on macrophage-mediated immunity, we depleted the macrophage population by injection of clodronate liposomes (61) at 30 hpf (or PBS liposomes as a negative control), leading to a significant depletion of the macrophage population (Fig. S8 available at https://doi.org/10.15131/shef.data.32630970). At 30 hpf, PR or DA *hif-1*α larvae were injected into the caudal vein with clodronate liposomes or PBS liposomes, followed by infection with 100 cfu *C. albicans* TT21-dTomato at 2 dpf. Both groups of PBS liposome-injected larvae had greater survival than their clodronate liposome, macrophage-depleted, siblings. DA *hif-1*α was host protective both with or without depletion of macrophages, with DA *hif-1*α + clodronate liposome larvae having greater survival than PR + clodronate liposome larvae (Fig. 6B; *P* < 0.0001), indicating that the host-protective effect of Hif-1α stabilization is not dependent on the presence of macrophages. Next, we injected one-cell stage zebrafish larvae with a *csf3r* morpholino (47) to deplete neutrophil numbers by over 60% in 2 dpf zebrafish larvae (Fig. S9 available at https://doi.org/10.15131/shef.data.32630970). *csf3r* morpholino injection led to a significant decrease in survival of 100 cfu *C. albicans*-infected larvae in both PR and DA *hif-1*α larvae (Fig. 6C). DA *hif-1*α *+ csf3*r larvae had lower survival compared to PR + *csf3r* larvae, showing a loss of the protective effect of Hif-1α stabilization in neutrophil-ablated larvae (Fig. 6C), indicating that the protective effect of Hif-1α stabilization is neutrophil-dependent.

**Fig 7:**
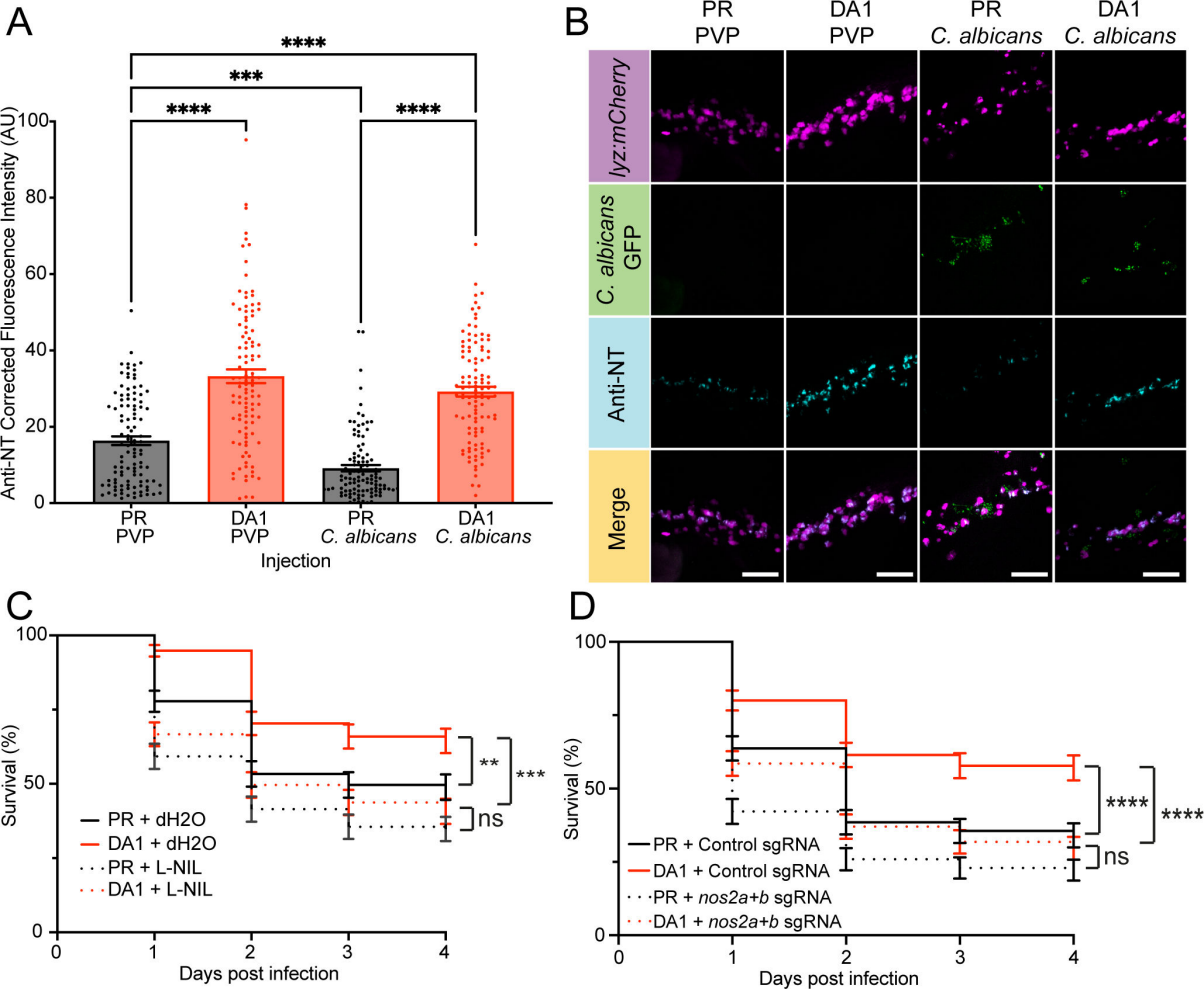
The host protective effect of Hif-1α stabilization was RNS-dependent. (**A**) Anti-nitrotyrosine fluorescence at 1 dpi in PR- and DA1-injected fish, following injection of PVP or 500 cfu *C. albicans* SN148 GFP. *N* = 108 neutrophils from 18 fish, obtained from three independent experiments. Error bars show SEM. Statistical significance determined by Kruskal-Wallis test, with Dunn’s multiple comparisons test. ***P < 0.001, ****P < 0.0001. (**B**) Representative images of 1 dpi Tg(*lyz:NTRmCherry*) zebrafish larvae injected with PR or DA1 and infected with 500 cfu *C. albicans* SN148 GFP or PVP. Scale bars = 50 µm. (**C**) Survival curve of 200 cfu *C. albicans* TT21-dTomato-infected zebrafish larvae following injection with PR or DA1, then treatment with dH_2_O or L-NIL. *N* = 135 fish, obtained from three independent experiments. Error bars show SEM. Statistical significance determined by Gehan-Breslow-Wilcoxon test, with Bonferroni correction. **P < 0.01, ***P < 0.001. (**D**) Survival curve of 200 cfu *C. albicans* TT21-dTomato-infected zebrafish larvae following injection with PR or DA1 and *tyr* or *nos2a*+*b* sgRNA. *N* = 135 fish, obtained from three independent experiments. Error bars show SEM. Statistical significance determined by Gehan-Breslow-Wilcoxon test, with Bonferroni correction. **P < 0.01, ***P < 0.001.

DA *hif-1*α did not alter the recruitment of neutrophils to the site of a local hindbrain *C. albicans* infection compared to PR-injected controls (Fig. S10 available at https://doi.org/10.15131/shef.data.32630970). Therefore, we hypothesized that the host protective effect of Hif-1α stabilization in *C. albicans* infection was due to increased neutrophil RNS production. DA *hif-1*α doubled the levels of nitrotyrosine compared to PR in mock-infected (PVP) larvae (Fig. 7A and B), similar to previous observations (31). While *C. albicans* reduced nitrotyrosine levels in PR control-injected larvae, DA *hif-1*α *C. albicans*-infected larvae had high levels of neutrophil nitrotyrosine compared to mock-infected (PVP) DA1 controls (Fig. 7A and B).

To demonstrate that the increase in RNS after Hif-1α stabilization is responsible for the host protective effect in *C. albicans* infection, we inhibited production of RNS both pharmacologically, using L-NIL, and genetically, using *nos2a + b* double CRISPR-Cas9 knockdown (“CRISPants” [48, 62]). L-NIL treatment did not impact the growth of *C. albicans in vitro* (Fig. S11 available at https://doi.org/10.15131/shef.data.32630970). Following infection with *C. albicans*, PR + L-NIL-treated larvae had reduced survival compared to PR + dH_2_O (drug solvent control)-treated larvae (Fig. 7C), indicating a role of RNS in *C. albicans* control. DA *hif-1*α + L-NIL-treated larvae did not have a significant survival advantage compared to either PR + dH_2_O or PR + L NIL groups (Fig. 7C), demonstrating that iNOS inhibition is sufficient to remove the host protective effect of Hif-1α stabilization. *Nos2a*+*b* double CRISPants were shown to inhibit nitrotyrosine production in mock-infected zebrafish (Fig. S12 available at https://doi.org/10.15131/shef.data.32630970). DA *hif-1*α with *nos2a*+*b* CRISPants no longer had increased survival following *C. albicans* infection compared to PR + *tyr* sgRNA (*tyrosinase* CRISPant control [48]) larvae, supporting that Nos2 is required for Hif-1α stabilization-dependent host protection (Fig. 7D). Together, these data demonstrate that Hif-1α stabilization is host-protective in *C. albicans* infection via a neutrophil- (Fig. 6) and RNS-dependent (Fig. 7) mechanism.

### Hif-1α stabilization has an additive host protective effect when used alongside antifungals

**Fig 8:**
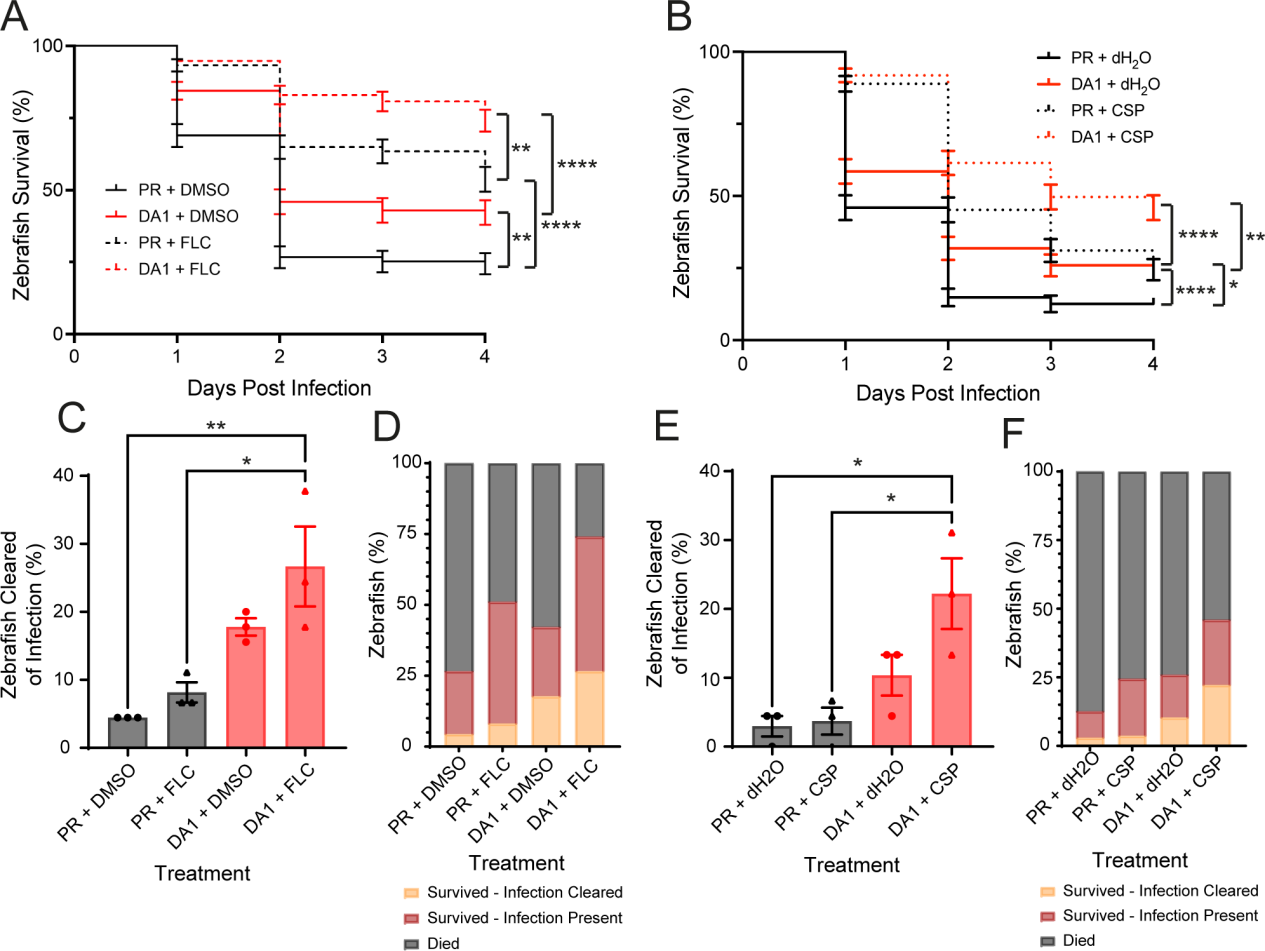
Hif-1α stabilization had an additive effect with antifungals. (**A**) Survival of *C. albicans* TT21-dTomato-infected zebrafish larvae following injection with dominant active *hif-1*α (DA1) or phenol red (PR) control. Larvae were treated with 5.0 µg/mL fluconazole or DMSO solvent control. Mortality was measured daily. *N* = 135 fish, obtained from three independent experiments. Error bars show SEM. Statistical significance was determined by Gehan-Breslow-Wilcoxon test with Bonferroni correction. **P < 0.01, ****P < 0.0001 (**B**) Survival of *C. albicans* TT21-dTomato-infected zebrafish larvae following injection with DA1 or PR control. Larvae were treated with 1.0 µg/mL caspofungin or dH_2_O solvent control. Mortality was measured daily. *N* = 135 fish, obtained from three independent experiments. Error bars show SEM. Statistical significance was determined by Gehan-Breslow-Wilcoxon test with Bonferroni correction. **P < 0.01, ****P < 0.0001. (**C**) Clearance of *C. albicans* infection at 4 dpi by zebrafish larvae following injection with DA1 or PR control and treatment with 5.0 µg/mL fluconazole or DMSO solvent control. *N* = 3 independent experiments, 135 fish per group. Error bars show Standard Deviation. Statistical significance was determined by one-way ANOVA, with Tukey’s multiple comparisons test. *P < 0.05, **P < 0.01 (**D**) Infection outcomes of *C. albicans* TT21-dTomato infected zebrafish larvae at 4 dpi, following injection with DA1 or PR and treatment with 5.0 µg/mL fluconazole or DMSO solvent control. *N* = 3 independent experiments, 135 fish per group. (**E**) Clearance of *C. albicans* infection at 4 dpi by zebrafish larvae following injection with DA1 or PR control and treatment with 1.0 µg/mL caspofungin or dH_2_O solvent control. *N* = 3 independent experiments, 135 fish per group. Error bars show standard deviation. Statistical significance was determined by one-way ANOVA, with Tukey’s multiple comparisons test. (**F**) Infection outcomes of *C. albicans* TT21-dTomato infected zebrafish larvae at 4 dpi, following injection with DA1 or PR and treatment with 1.0 µg/mL caspofungin or dH_2_O solvent control. *N* = 3 independent experiments, 135 fish per group.

We next investigated the potential of Hif-1α stabilization to act as an adjunctive therapy alongside established antifungals. At 1 dpf, PR/DA *hif-1*α larvae were injected into the caudal vein with 500 cfu *C. albicans* TT21-dTomato and then treated by immersion with 5.0 μg/mL fluconazole or 1.0 µg/mL caspofungin. Both fluconazole (Fig. S13 available at https://doi.org/10.15131/shef.data.32630970) and caspofungin (Fig. S14 available at https://doi.org/10.15131/shef.data.32630970) treatment had dose-dependent effects on zebrafish larval survival post*-C. albicans* infection. Fluconazole significantly increased host survival in both PR and DA *hif-1*α larvae (Fig. 8A). DA *hif-1*α + fluconazole had increased survival compared to PR + fluconazole and DA *hif-1*α + DMSO. Combination of DA *hif-1*α + caspofungin similarly increased survival compared to PR + caspofungin (Fig. 8B) and DA *hif-1*α + dH_2_O (Fig. 8B), demonstrating that the combination of Hif-1α stabilization with fluconazole or caspofungin has an additive effect on survival.

Surviving larvae were imaged at 4 dpi to quantify the clearance of *C. albicans* infection (Fig. 8C through F); 27% of DA *hif-1*α + fluconazole larvae had completely cleared infection at 4 dpi (Fig. 8C and D), greater than both PR alone (4%) and PR +fluconazole (8%). In total, 22% of larvae treated with DA *hif-1*α + caspofungin cleared infection (Fig. 8E and F) compared to 3% of PR-treated larvae and 4% of larvae treated with PR and caspofungin. Hence, Hif-1α stabilization also has an additive effect on clearance when combined with fluconazole or caspofungin.

## DISCUSSION

The increasing prevalence of antifungal resistance worldwide highlights the importance of discovering new antifungal targets or host-directed therapies capable of improving disease outcomes. Here, we have shown that *Candida* species suppression of neutrophil RNS is an important factor in their virulence *in vivo,* and that restoration of neutrophil RNS represents a potential host-directed therapy for difficult-to-treat *C. albicans* infection.

We found that *C. albicans* can suppress RNS production in zebrafish neutrophils *in vivo* and in human primary neutrophils *in vitro. C. albicans* has been shown to suppress RNS production in murine bone marrow-derived macrophages *in vitro* (19), which is supported by a variety of sources demonstrating the suppression of ROS by *C. albicans in vitro* (18, 63). While previous publications have demonstrated RNS suppression in macrophages, our data demonstrate an effect in neutrophils in a live tissue setting. Human macrophages are poor RNS producers compared to mouse macrophages (64, 65), and *C. albicans* is primarily controlled by neutrophils in human disease (66). Therefore, we chose to dissect the mechanisms behind RNS suppression in neutrophils in the tractable *in vivo* zebrafish model.

Here, we identify that RNS suppression is partially dependent on live, hypha-forming *C. albicans*. The hyphal morphotype is highly associated with a shift in transcription and increased virulence (67, 68), which may trigger the expression of protein(s) necessary for RNS suppression by *C. albicans*. Proteomic analysis of supernatant from hyphal *C. albicans in vitro* identified 301 secreted proteins specific to hyphae (57). Further examination and characterization of these proteins could point toward candidates involved in active RNS suppression. However, RNS suppression was still observed in non-hypha-forming, non-albicans *Candida* species isolates (*C. glabrata, C. parapsilosis,* and *C. guilliermondii*), suggesting that the presence of hyphae is not essential for RNS suppression.

*C. albicans*-derived arginase also contributed to the suppression of neutrophil RNS production. CAR1 is the only known arginase gene in the *C. albicans* genome, encoding a cytosolic arginase (43). CAR1 has been shown to be important in the hyphal morphogenesis response to arginine and for virulence in murine infection models. Our findings indicate fungal arginase could be interfering with the host arginase-iNOS balance, resulting in reduced iNOS activity and RNS suppression. Contrary to this, *C. albicans car1*Δ infection in a murine macrophage model did not influence RNS synthesis (43). As CAR1 is not considered a secreted protein, residing in the cytosol, the mechanism of how it would interact with neutrophil RNS is unclear. However, other immunogenic *C. albicans* enzymes have been shown to be released into the extracellular space during stress or death (69), which may be a potential point of interaction between CAR1 and neutrophil RNS production during *in vivo* pathogenesis. The modest decrease in neutrophil RNS production after infection with the CAR1 mutant suggests that *C. albicans* arginase production is not the sole RNS-reducing mechanism. A caveat to this experiment is that the complemented strain of the *car1*Δ *C. albicans* strain was not used; therefore, we do not control for any artefactual chromosomal changes that may have occurred during *car1*Δ deletion. Heat-killed *C. albicans* was able to partially suppress neutrophil RNS, suggesting that mechanisms may be partially passive. The presence of chitin may account for the partial suppression of RNS by heat-killed *C. albicans* that we observed, with *C. albicans* chitin shown to induce host arginase-1 activity, decreasing RNS production (70). Our findings suggest that multiple, additive mechanisms of neutrophil RNS suppression by *C. albicans* may underlie their success at the large overall suppression of neutrophil RNS observed. There are several potential *C. albicans* candidate molecules that may be involved in this process. *C. albicans* SOD5 has been associated with degradation of antimicrobial ROS *in vitro* and is found on the cell surface allowing interaction with the extracellular environment (63). *C. albicans sod5*Δ/Δ have decreased viability in the presence of macrophages (63). *C. albicans YHB1* is able to detoxify RNS (71); however, it is thought to be non-secreted with a cytosolic and mitochondrial localization. Hence, similar to CAR1, the mechanism of extracellular interaction is as yet unclear. *C. albicans* strains with deleted YHB1 are hypersensitive to RNS and are hypofilamentous (71). Collette et al. identified an unknown, small secreted molecule from *C. albicans* capable of suppressing RNS (19). Interestingly, RNS inhibition in mouse macrophages required direct C. albicans interaction, with RNS inhibition lost using transwell plates of 0.4 μm pores. However, the inhibitory molecule(s) was secreted, as co-culture supernatant was able to decrease RNS levels (19). They identified that the compound was small (less than 3 kDa in size), aqueous and heat-stable, a profile which fits the physical characteristics of many tissue culture media ingredients, impeding identification using standard *in vitro* methods. We have yet to elucidate whether RNS suppression is a fully active process that has evolved to evade immune cells, either incidentally via evolution of predation in the soil by amoebae (72) or as an intentional immune evasion strategy, or whether it is partially a passive process. Further investigation could aim to establish which are the most important mechanisms and effector proteins involved in RNS suppression in neutrophils.

Neutrophil RNS suppression was observed in infection with various *C. albicans* laboratory strains and clinical isolates alongside non-albicans *Candida* species clinical isolates. This suggests that RNS suppression could be conserved widely across clinically relevant *Candida* spp. Importantly, *Candida* species virulence correlated with neutrophil RNS suppression *in vivo,* indicating that neutrophil RNS suppression may be a virulence factor. Virulence is determined by a wide range of factors, not solely neutrophil RNS suppression (73), any of which could be differentially regulated in these *Candida* species isolates. With differences in virulence, there may be differences in disease pathogenesis that could influence RNS suppression that are challenging to measure without fluorescent labeling; for example, different fungal burden may have an impact on the level of RNS at the time points measured. It was interesting to note that the emerging human pathogen *C. auris* was able to efficiently suppress neutrophil RNS in zebrafish, despite only being identified as a human pathogen since 2009 (74), suggesting that this immune evasion mechanism may have evolved in the environment (75). Suppression of neutrophil RNS by environmental strains may act as a useful proxy for determining whether *Candida* spp. or strains have the potential to be virulent in humans.

We found that upregulation of RNS, via Hif-1α stabilization, has a protective effect in *C. albicans* infection in zebrafish larvae, via a neutrophil-mediated, RNS-dependent mechanism. Neutrophils are the primary innate immune cell responsible for killing *Candida* spp. (10). Neutrophil activity against *Candida* spp. is considered strong, as evidenced by high mortality in neutropenic patients (76). They are able to perform intracellular killing (66) alongside extracellular killing via NET (neutrophil extracellular trap) release (77). However, our data indicate not only suppression of a protective neutrophil mechanism but also suggest that the neutrophil response to *Candida* spp. can be improved. Neutrophil RNS suppression is therefore an important mechanism of *C. albicans* pathogenesis that, if restored, could represent an exciting therapeutic opportunity. RNS is well characterized as being candidacidal (12, 13, 15, 78) and enhances NET release (79). NO-releasing nanoparticles inhibited the growth of *C. albicans in vitro* and *in vivo* (80). Hence, upregulation of neutrophil RNS production is a potential mechanism that could be targeted therapeutically to improve immune candidacidal activity. We have previously demonstrated that Hif-1α stabilization protects against *M. marinum* infection in a similar manner (31), suggesting that Hif-1α stabilization could offer a protective effect against multiple and different groups of pathogens (bacteria and fungi) where neutrophil-mediated immune responses are dampened during pathogenesis.

We further showed that Hif-1α stabilization had an additive effect on survival and clearance when combined with traditional antifungals. Early fungicidal activity by neutrophils prior to hyphal growth is vital for subsequent clearance and resolution of *C. albicans* infection, with early phagocytosis a strong prognostic indicator of survival (81, 82). As a primarily fungistatic agent (83), fluconazole may help to restrict initial growth of *C. albicans*, allowing neutrophils with increased RNS production to kill *C. albicans,* resulting in greater survival and clearance. Conversely, caspofungin predominantly has a fungicidal effect (84, 85), although increased neutrophil RNS production therapeutically may also facilitate earlier killing of *C. albicans* in combination and allow lower doses of these poorly tolerated drugs for shorter treatment periods. Exactly when and how combination therapy should be administered to patients requires follow-on studies.

Our data show that *Candida* spp. can efficiently suppress neutrophil RNS as a virulence mechanism and that Hif-1α stabilization is able to overcome this, conferring a neutrophil-mediated, RNS-dependent protective effect *in vivo*. Neutrophil RNS restoration has the potential as an adjunctive host-directed therapeutic strategy to aid in the treatment of *Candida* species infections.

## References

[1] Witherden EA, Shoaie S, Hall RA, Moyes DL. 2017. The human mucosal mycobiome and fungal community interactions. J Fungi (Basel) 3:56. doi:10.3390/jof3040056 29371572 PMC5753158

[2] Flores-Maldonado OE, Becerril-García MA, Treviño-Rangel R de J, Rodríguez-Gámez LA, González GM. 2019. Neonatal invasive candidiasis: a poorly understood entity. RMU 21. doi:10.24875/RMU.18000031

[3] Pellon A, Sadeghi Nasab SD, Moyes DL. 2020. New insights in *Candida albicans* innate immunity at the mucosa: toxins, epithelium, metabolism, and beyond. Front Cell Infect Microbiol 10:81. doi:10.3389/fcimb.2020.00081 32195196 PMC7062647

[4] Bongomin F, Gago S, Oladele R, Denning D. 2017. Global and multi-national prevalence of fungal diseases—estimate precision. J Fungi 3:57. doi:10.3390/jof3040057 PMC5753159 29371573

[5] WHO. 2022. WHO fungal priority pathogens list to guide research, development and public health action. World Health Organization, Geneva.

[6] Lamoth F, Lockhart SR, Berkow EL, Calandra T. 2018. Changes in the epidemiological landscape of invasive candidiasis. J Antimicrob Chemother 73:i4–i13. doi:10.1093/jac/dkx444 29304207 PMC11931512

[7] Almeida F, Rodrigues ML, Coelho C. 2019. The still underestimated problem of fungal diseases worldwide. Front Microbiol 10:214. doi:10.3389/fmicb.2019.00214 30809213 PMC6379264

[8] Burgess TB, Condliffe AM, Elks PM. 2022. A fun-guide to innate immune responses to fungal infections. J Fungi (Basel) 8:805. doi:10.3390/jof8080805 36012793 PMC9409918

[9] Uzun O, Ascioglu S, Anaissie EJ, Rex JH. 2001. Risk factors and predictors of outcome in patients with cancer and breakthrough candidemia. Clin Infect Dis 32:1713–1717. doi:10.1086/320757 11360213

[10] Brown GD. 2011. Innate antifungal immunity: the key role of phagocytes. Annu Rev Immunol 29:1–21. doi:10.1146/annurev-immunol-030409-101229 20936972 PMC3434799

[11] Winterbourn CC, Hampton MB, Livesey JH, Kettle AJ. 2006. Modeling the reactions of superoxide and myeloperoxidase in the neutrophil phagosome: implications for microbial killing. J Biol Chem 281:39860–39869. doi:10.1074/jbc.M605898200 17074761

[12] Rementería A, García-Tobalina R, Sevilla MJ. 1995. Nitric oxide-dependent killing of *Candida albicans* by murine peritoneal cells during an experimental infection. FEMS Immunol Med Microbiol 11:157–162. doi:10.1111/j.1574-695X.1995.tb00112.x 7581266

[13] Vazquez-Torres A, Jones-Carson J, Warner T, Balish E. 1995. Nitric oxide enhances resistance of SCID mice to mucosal candidiasis. J Infect Dis 172:192–198. doi:10.1093/infdis/172.1.192 7541062

[14] Vazquez-Torres A, Jones-Carson J, Balish E. 1996. Peroxynitrite contributes to the candidacidal activity of nitric oxide-producing macrophages. Infect Immun 64:3127–3133. doi:10.1128/iai.64.8.3127-3133.1996 8757843 PMC174197

[15] Navarathna DH, Lionakis MS, Roberts DD. 2019. Endothelial nitric oxide synthase limits host immunity to control disseminated *Candida albicans* infections in mice. PLOS One 14:e0223919. doi:10.1371/journal.pone.0223919 31671151 PMC6822743

[16] Bansal V, Ochoa JB. 2003. Arginine availability, arginase, and the immune response. Curr Opin Clin Nutr Metab Care 6:223–228. doi:10.1097/00075197-200303000-00012 12589193

[17] Luo S, Skerka C, Kurzai O, Zipfel PF. 2013. Complement and innate immune evasion strategies of the human pathogenic fungus *Candida albicans*. Mol Immunol 56:161–169. doi:10.1016/j.molimm.2013.05.218 23809232

[18] Wellington M, Dolan K, Krysan DJ. 2009. Live *Candida albicans* suppresses production of reactive oxygen species in phagocytes. Infect Immun 77:405–413. doi:10.1128/IAI.00860-08 18981256 PMC2612242

[19] Collette JR, Zhou H, Lorenz MC. 2014. *Candida albicans* suppresses nitric oxide generation from macrophages via a secreted molecule. PLoS One 9:e96203. doi:10.1371/journal.pone.0096203 24755669 PMC3995984

[20] Henry KM, Loynes CA, Whyte MKB, Renshaw SA. 2013. Zebrafish as a model for the study of neutrophil biology. J Leukoc Biol 94:633–642. doi:10.1189/jlb.1112594 23463724

[21] Speirs ZC, Loynes CA, Mathiessen H, Elks PM, Renshaw SA, Jørgensen L von G. 2024. What can we learn about fish neutrophil and macrophage response to immune challenge from studies in zebrafish. Fish & Shellfish Immunology 148:109490. doi:10.1016/j.fsi.2024.109490 38471626

[22] Lam SH, Chua HL, Gong Z, Lam TJ, Sin YM. 2004. Development and maturation of the immune system in zebrafish, *Danio rerio*: a gene expression profiling, *in situ* hybridization and immunological study. Dev Compar Immunol 28:9–28. doi:10.1016/S0145-305X(03)00103-4 12962979

[23] Renshaw SA, Loynes CA, Trushell DMI, Elworthy S, Ingham PW, Whyte MKB. 2006. A transgenic zebrafish model of neutrophilic inflammation. Blood 108:3976–3978. doi:10.1182/blood-2006-05-024075 16926288

[24] Chao C-C, Hsu P-C, Jen C-F, Chen I-H, Wang C-H, Chan H-C, Tsai P-W, Tung K-C, Wang C-H, Lan C-Y, et al.. 2010. Zebrafish as a model host for *Candida albicans* infection. Infect Immun 78:2512–2521. doi:10.1128/IAI.01293-09 20308295 PMC2876552

[25] Rosowski EE, Knox BP, Archambault LS, Huttenlocher A, Keller NP, Wheeler RT, Davis JM. 2018. The zebrafish as a model host for invasive fungal infections. J Fungi (Basel) 4:136. doi:10.3390/jof4040136 30551557 PMC6308935

[26] Archambault LS, Trzilova D, Gonia S, Gale C, Wheeler RT. 2019. Intravital imaging reveals divergent cytokine and cellular immune responses to *Candida albicans* and *Candida parapsilosis*. mBio 10:e00266-19. doi:10.1128/mBio.00266-19 31088918 PMC6520444

[27] Blair BA, Bragdon E, Dhillon G, Baker N, Stasiak L, Muthig M, Miramon P, Lorenz MC, Wheeler RT. 2025. Forward genetic screen in zebrafish identifies new fungal regulators that limit host-protective *Candida*-innate immune interaction. mBio 16:e00529-25. doi:10.1128/mbio.00529-25 40172223 PMC12077120

[28] Gratacap RL, Scherer AK, Seman BG, Wheeler RT. 2017. Control of mucosal candidiasis in the zebrafish swim bladder depends on neutrophils that block filament invasion and drive extracellular-trap production. Infect Immun 85:e00276-17. doi:10.1128/IAI.00276-17 28607100 PMC5563567

[29] Seman BG, Moore JL, Scherer AK, Blair BA, Manandhar S, Jones JM, Wheeler RT. 2018. Yeast and filaments have specialized, independent activities in a zebrafish model of *Candida albicans* infection. Infect Immun 86:e00415-18. doi:10.1128/IAI.00415-18 30037799 PMC6204735

[30] Christou S, Evans S, Knafler H, Rooij IS, Ayscough KR, Johnston SA. 2026. *Candida albicans* cells lacking AP-2 have defective hyphae and are avirulent despite increased host uptake and intracellular proliferation in macrophages. Mol Microbiol 125:1–12. doi:10.1111/mmi.70032 41178127 PMC12763539

[31] Elks PM, Brizee S, van der Vaart M, Walmsley SR, van Eeden FJ, Renshaw SA, Meijer AH. 2013. Hypoxia inducible factor signaling modulates susceptibility to mycobacterial infection via a nitric oxide dependent mechanism. PLoS Pathog 9:e1003789. doi:10.1371/journal.ppat.1003789 24367256 PMC3868520

[32] McGettrick AF, O’Neill LAJ. 2020. The role of HIF in immunity and inflammation. Cell Metab 32:524–536. doi:10.1016/j.cmet.2020.08.002 32853548

[33] Peyssonnaux C, Datta V, Cramer T, Doedens A, Theodorakis EA, Gallo RL, Hurtado-Ziola N, Nizet V, Johnson RS. 2005. HIF-1alpha expression regulates the bactericidal capacity of phagocytes. J Clin Invest 115:1806–1815. doi:10.1172/JCI23865 16007254 PMC1159132

[34] Jantsch J, Wiese M, Schödel J, Castiglione K, Gläsner J, Kolbe S, Mole D, Schleicher U, Eckardt K-U, Hensel M, et al.. 2011. Toll-like receptor activation and hypoxia use distinct signaling pathways to stabilize hypoxia-inducible factor 1α (HIF1A) and result in differential HIF1A-dependent gene expression. J Leukoc Biol 90:551–562. doi:10.1189/jlb.1210683 21685248

[35] Krzywinska E, Stockmann C. 2018. Hypoxia, metabolism and immune cell function. Biomedicines 6:56. doi:10.3390/biomedicines6020056 29762526 PMC6027519

[36] Hammond FR, Lewis A, Elks PM. 2020. If it’s not one thing, HIF’s another: immunoregulation by hypoxia inducible factors in disease. FEBS J 287:3907–3916. doi:10.1111/febs.15476 32633061 PMC7362030

[37] Buchan KD, Prajsnar TK, Ogryzko NV, de Jong NWM, van Gent M, Kolata J, Foster SJ, van Strijp JAG, Renshaw SA. 2019. A transgenic zebrafish line for in vivo visualisation of neutrophil myeloperoxidase. PLoS One 14:e0215592. doi:10.1371/journal.pone.0215592 31002727 PMC6474608

[38] Bernut A, Dupont C, Ogryzko NV, Neyret A, Herrmann J-L, Floto RA, Renshaw SA, Kremer L. 2019. CFTR Protects against *Mycobacterium abscessus* infection by fine-tuning host oxidative defenses. Cell Rep 26:1828–1840. doi:10.1016/j.celrep.2019.01.071 30759393 PMC7618368

[39] Hammond FR, Lewis A, Speirs ZC, Anderson HE, Sipka T, Williams LG, Nguyen-Chi M, Meijer AH, Wiegertjes GF, Elks PM. 2023. An arginase 2 promoter transgenic line illuminates immune cell polarisation in zebrafish. Dis Model Mech 16:dmm049966. doi:10.1242/dmm.049966 37078586 PMC10163355

[40] Santhakumar K, Judson EC, Elks PM, McKee S, Elworthy S, van Rooijen E, Walmsley SS, Renshaw SA, Cross SS, van Eeden FJM. 2012. A zebrafish model to study and therapeutically manipulate hypoxia signaling in tumorigenesis. Cancer Res 72:4017–4027. doi:10.1158/0008-5472.CAN-11-3148 22665266

[41] Panpetch W, Somboonna N, Bulan DE, Issara-Amphorn J, Finkelman M, Worasilchai N, Chindamporn A, Palaga T, Tumwasorn S, Leelahavanichkul A. 2017. Oral administration of live- or heat-killed *Candida albicans* worsened cecal ligation and puncture sepsis in a murine model possibly due to an increased serum (1→3)-β-D-glucan. PLoS One 12:e0181439. doi:10.1371/journal.pone.0181439 28750040 PMC5531434

[42] Gillum AM, Tsay EY, Kirsch DR. 1984. Isolation of the *Candida albicans* gene for orotidine-5’-phosphate decarboxylase by complementation of *S. cerevisiae* ura3 and *E. coli* pyrF mutations. Mol Gen Genet 198:179–182. doi:10.1007/BF00328721 6394964

[43] Schaefer K, Wagener J, Ames RM, Christou S, MacCallum DM, Bates S, Gow NAR. 2020. Three related enzymes in *Candida albicans* achieve arginine- and agmatine-dependent metabolism that is essential for growth and fungal virulence. mBio 11:e01845-20. doi:10.1128/mBio.01845-20 32788384 PMC7439472

[44] Knafler HC, Smaczynska-de Rooij II, Walker LA, Lee KK, Gow NAR, Ayscough KR. 2019. AP-2-Dependent endocytic recycling of the chitin synthase chs3 regulates polarized growth in *Candida albicans*. mBio 10:e02421-18. doi:10.1128/mBio.02421-18 30890602 PMC6426607

[45] Prajsnar TK, Cunliffe VT, Foster SJ, Renshaw SA. 2008. A novel vertebrate model of *Staphylococcus aureus* infection reveals phagocyte-dependent resistance of zebrafish to non-host specialized pathogens. Cell Microbiol 10:2312–2325. doi:10.1111/j.1462-5822.2008.01213.x 18715285

[46] Elks PM, van Eeden FJ, Dixon G, Wang X, Reyes-Aldasoro CC, Ingham PW, Whyte MKB, Walmsley SR, Renshaw SA. 2011. Activation of hypoxia-inducible factor-1α (Hif-1α) delays inflammation resolution by reducing neutrophil apoptosis and reverse migration in a zebrafish inflammation model. Blood 118:712–722. doi:10.1182/blood-2010-12-324186 21555741

[47] Ellett F, Pase L, Hayman JW, Andrianopoulos A, Lieschke GJ. 2011. Mpeg1 promoter transgenes direct macrophage-lineage expression in zebrafish. Blood 117:e49–56. doi:10.1182/blood-2010-10-314120 21084707 PMC3056479

[48] Isles HM, Herman KD, Robertson AL, Loynes CA, Prince LR, Elks PM, Renshaw SA. 2019. The CXCL12/CXCR4 Signaling axis retains neutrophils at inflammatory sites in zebrafish. Front Immunol 10. doi:10.3389/fimmu.2019.01784 PMC6684839 31417560

[49] Lewis A, Elks PM. 2019. Hypoxia Induces macrophage tnfa expression via cyclooxygenase and prostaglandin E2 *in vivo*. Front Immunol 10:2321. doi:10.3389/fimmu.2019.02321 31611882 PMC6776637

[50] Elks PM, van der Vaart M, van Hensbergen V, Schutz E, Redd MJ, Murayama E, Spaink HP, Meijer AH. 2014. Mycobacteria counteract a TLR-mediated nitrosative defense mechanism in a zebrafish infection model. PLoS One 9:e100928. doi:10.1371/journal.pone.0100928 24967596 PMC4072692

[51] Burgess A, Vigneron S, Brioudes E, Labbé J-C, Lorca T, Castro A. 2010. Loss of human greatwall results in G2 arrest and multiple mitotic defects due to deregulation of the cyclin B-Cdc2/PP2A balance. Proc Natl Acad Sci USA 107:12564–12569. doi:10.1073/pnas.0914191107 20538976 PMC2906566

[52] Thrikawala S, Niu M, Keller NP, Rosowski EE. 2022. Cyclooxygenase production of PGE2 promotes phagocyte control of *A. fumigatus* hyphal growth in larval zebrafish. PLOS Pathog 18:e1010040. doi:10.1371/journal.ppat.1010040 35333905 PMC8986117

[53] Herman KD, Rahman A, Prince LR. 2020. Isolation and high throughput flow cytometric apoptosis assay of human neutrophils to enable compound library screening. Bio Protoc 10:e3640. doi:10.21769/BioProtoc.3640 PMC7842575 33659311

[54] Iracane E, Arias-Sardá C, Maufrais C, Ene IV, d’Enfert C, Buscaino A. 2024. Identification of an active RNAi pathway in *Candida albicans*. Proc Natl Acad Sci USA 121:e2315926121. doi:10.1073/pnas.2315926121 38625945 PMC11047096

[55] Nantel A, Dignard D, Bachewich C, Harcus D, Marcil A, Bouin A-P, Sensen CW, Hogues H, van het Hoog M, Gordon P, et al.. 2002. Transcription profiling of *Candida albicans* cells undergoing the yeast-to-hyphal transition. Mol Biol Cell 13:3452–3465. doi:10.1091/mbc.e02-05-0272 12388749 PMC129958

[56] Höfs S, Mogavero S, Hube B. 2016. Interaction of *Candida albicans* with host cells: virulence factors, host defense, escape strategies, and the microbiota. J Microbiol 54:149–169. doi:10.1007/s12275-016-5514-0 26920876

[57] Vaz C, Pitarch A, Gómez-Molero E, Amador-García A, Weig M, Bader O, Monteoliva L, Gil C. 2021. Mass spectrometry-based proteomic and immunoproteomic analyses of the *Candida albicans* hyphal secretome reveal diagnostic biomarker candidates for invasive candidiasis. J Fungi 7:501. doi:10.3390/jof7070501 PMC8306665 34201883

[58] Garcia-Rubio R, de Oliveira HC, Rivera J, Trevijano-Contador N. 2019. The fungal cell wall: Candida, cryptococcus, and *Aspergillus* species. Front Microbiol 10:2993. doi:10.3389/fmicb.2019.02993 31993032 PMC6962315

[59] Desai JV, Lionakis MS. 2018. The role of neutrophils in host defense against invasive fungal infections. Curr Clin Microbiol Rep 5:181–189. doi:10.1007/s40588-018-0098-6 31552161 PMC6758935

[60] Brothers KM, Gratacap RL, Barker SE, Newman ZR, Norum A, Wheeler RT. 2013. NADPH oxidase-driven phagocyte recruitment controls *Candida albicans* filamentous growth and prevents mortality. PLoS Pathog 9:e1003634. doi:10.1371/journal.ppat.1003634 24098114 PMC3789746

[61] van Rooijen N, Hendrikx E. 2010. Liposomes for specific depletion of macrophages from organs and tissues. Methods Mol Biol 605:189–203. doi:10.1007/978-1-60327-360-2_13 20072882

[62] Moore WM, Webber RK, Jerome GM, Tjoeng FS, Misko TP, Currie MG. 1994. L-N6-(1-iminoethyl)lysine: a selective inhibitor of inducible nitric oxide synthase. J Med Chem 37:3886–3888. doi:10.1021/jm00049a007 7525961

[63] Frohner IE, Bourgeois C, Yatsyk K, Majer O, Kuchler K. 2009. *Candida albicans* cell surface superoxide dismutases degrade host-derived reactive oxygen species to escape innate immune surveillance. Mol Microbiol 71:240–252. doi:10.1111/j.1365-2958.2008.06528.x 19019164 PMC2713856

[64] Weinberg J, Misukonis M, Shami P, Mason S, Sauls D, Dittman W, Wood E, Smith G, McDonald B, Bachus K. 1995. Human mononuclear phagocyte inducible nitric oxide synthase (iNOS): analysis of iNOS mRNA, iNOS protein, biopterin, and nitric oxide production by blood monocytes and peritoneal macrophages. Blood 86:1184–1195. doi:10.1182/blood.V86.3.1184.1184 7542498

[65] Muijsers RBR, ten Hacken NHT, Van Ark I, Folkerts G, Nijkamp FP, Postma DS. 2001. L-Arginine is not the limiting factor for nitric oxide synthesis by human alveolar macrophages *in vitro*. Eur Respir J 18:667–671. doi:10.1183/09031936.01.00101301 11716172

[66] Gazendam RP, van de Geer A, Roos D, van den Berg TK, Kuijpers TW. 2016. How neutrophils kill fungi. Immunol Rev 273:299–311. doi:10.1111/imr.12454 27558342

[67] Sudbery PE. 2011. Growth of *Candida albicans hyphae*. Nat Rev Microbiol 9:737–748. doi:10.1038/nrmicro2636 21844880

[68] Desai JV. 2018. *Candida albicans hyphae*: from growth initiation to invasion. J Fungi (Basel) 4:10. doi:10.3390/jof4010010 29371503 PMC5872313

[69] De Cesare GB, Hafez A, Stead D, Llorens C, Munro CA. 2022. Biomarkers of caspofungin resistance in *Candida albicans* isolates: a proteomic approach. Virulence 13:1005–1018. doi:10.1080/21505594.2022.2081291 35730400 PMC9225221

[70] Wagener J, MacCallum DM, Brown GD, Gow NAR. 2017. *Candida albicans* chitin increases arginase-1 activity in human macrophages. mBio 8:e01820-16. doi:10.1128/mBio.01820-16 PMC5263244 28119468

[71] Hromatka BS, Noble SM, Johnson AD. 2005. Transcriptional response of *Candida albicans* to nitric oxide and the role of the YHB1 gene in nitrosative stress and virulence. Mol Biol Cell 16:4814–4826. doi:10.1091/mbc.e05-05-0435 16030247 PMC1237085

[72] Amsri A, Pruksaphon K, Thammasit P, Nosanchuk JD, Youngchim S. 2024. Adaptation to an amoeba host drives selection of virulence-associated traits and genetic variation in saprotrophic *Candida albicans*. Front Cell Infect Microbiol 14:1367656. doi:10.3389/fcimb.2024.1367656 38550616 PMC10976851

[73] Ghannoum MA, Abu-Elteen KH. 1990. Pathogenicity determinants of *Candida*. Mycoses 33:265–282. doi:10.1111/myc.1990.33.6.265 2259368

[74] Lockhart SR, Etienne KA, Vallabhaneni S, Farooqi J, Chowdhary A, Govender NP, Colombo AL, Calvo B, Cuomo CA, Desjardins CA, et al.. 2017. Simultaneous emergence of multidrug-resistant *Candida auris* on 3 continents confirmed by whole-genome sequencing and epidemiological analyses. CLINID 64:134–140. doi:10.1093/cid/ciw691 PMC5215215 27988485

[75] Casadevall A, Kontoyiannis DP, Robert V. 2019. On the emergence of *Candida auris*: climate change, azoles, swamps, and birds. mBio 10:e01397-19. doi:10.1128/mBio.01397-19 31337723 PMC6650554

[76] Herbrecht R, Neuville S, Letscher-Bru V, Natarajan-Am?? S, Lortholary O. 2000. Fungal infections in patients with neutropenia. Drugs Aging 17:339–351. doi:10.2165/00002512-200017050-00002 11190415

[77] Urban CF, Reichard U, Brinkmann V, Zychlinsky A. 2006. Neutrophil extracellular traps capture and kill *Candida albicans* yeast and hyphal forms. Cell Microbiol 8:668–676. doi:10.1111/j.1462-5822.2005.00659.x 16548892

[78] Tillmann A, Gow NAR, Brown AJP. 2011. Nitric oxide and nitrosative stress tolerance in yeast. Biochem Soc Trans 39:219–223. doi:10.1042/BST0390219 21265777 PMC3615668

[79] Manda-Handzlik A, Bystrzycka W, Cieloch A, Glodkowska-Mrowka E, Jankowska-Steifer E, Heropolitanska-Pliszka E, Skrobot A, Muchowicz A, Ciepiela O, Wachowska M, et al.. 2020. Nitric oxide and peroxynitrite trigger and enhance release of neutrophil extracellular traps. Cell Mol Life Sci 77:3059–3075. doi:10.1007/s00018-019-03331-x 31650185 PMC7366602

[80] Macherla C, Sanchez DA, Ahmadi MS, Vellozzi EM, Friedman AJ, Nosanchuk JD, Martinez LR. 2012. Nitric oxide releasing nanoparticles for treatment of *Candida albicans* burn infections. Front Microbiol 3:193. doi:10.3389/fmicb.2012.00193 22701111 PMC3370663

[81] Bergeron AC, Barker SE, Brothers KM, Prasad BC, Wheeler RT. 2017. Polyclonal anti-*Candida* antibody improves phagocytosis and overall outcome in zebrafish model of disseminated candidiasis. Dev Compar Immunol 68:69–78. doi:10.1016/j.dci.2016.11.017 PMC6700731 27884707

[82] Zhu W, Zhang H, Dong Q, Song H, Zhao L. 2023. Dual wave of neutrophil recruitment determines the outcome of *C. albicans* infection. Front Cell Infect Microbiol 13. doi:10.3389/fcimb.2023.1239593 PMC10364056 37492529

[83] Vasicek EM, Berkow EL, Bruno VM, Mitchell AP, Wiederhold NP, Barker KS, Rogers PD. 2014. Disruption of the transcriptional regulator cas5 results in enhanced killing of *Candida albicans* by fluconazole. Antimicrob Agents Chemother 58:6807–6818. doi:10.1128/AAC.00064-14 25182640 PMC4249418

[84] Letscher-Bru V, Herbrecht R. 2003. Caspofungin: the first representative of a new antifungal class. J Antimicrob Chemother 51:513–521. doi:10.1093/jac/dkg117 12615851

[85] Szymański M, Chmielewska S, Czyżewska U, Malinowska M, Tylicki A. 2022. Echinocandins - structure, mechanism of action and use in antifungal therapy. J Enzyme Inhib Med Chem 37:876–894. doi:10.1080/14756366.2022.2050224 35296203 PMC8933026

